# A systematic review of interventions for reducing heavy episodic drinking in sub-Saharan African settings

**DOI:** 10.1371/journal.pone.0242678

**Published:** 2020-12-01

**Authors:** Katelyn M. Sileo, Amanda P. Miller, Tina A. Huynh, Susan M. Kiene

**Affiliations:** 1 Department of Public Health, University of Texas at San Antonio, San Antonio, Texas, United States of America; 2 Division of Epidemiology and Biostatistics, San Diego State University School of Public Health, San Diego, California, United States of America; 3 Center for Interdisciplinary Research on AIDS (CIRA), Yale University, New Haven, Connecticut, United States of America; 4 Division of Infectious Disease and Global Public Health, Department of Medicine, The University of California, San Diego, La Jolla, California, United States of America; Augusta University, UNITED STATES

## Abstract

**Objective:**

Assess the effect of non-pharmacological alcohol interventions on reducing heavy episodic drinking (HED) outcomes in sub-Saharan Africa.

**Methods:**

A systematic review of the available literature through August 19, 2020 was conducted. Randomized and non-randomized controlled trials testing non-pharmacological interventions on alcohol consumption in sub-Saharan Africa were eligible for inclusion. Eligible outcomes included measures of HED/binge drinking, and measures indicative of this pattern of drinking, such as high blood alcohol concentration or frequency of intoxication. Three authors extracted and reconciled relevant data and assessed risk of bias. The review protocol is available on PROSPERO (registration number: CRD42019094509). The Cochrane Handbook recommendations for the review of interventions and the Preferred Reporting Items for Systematic Reviews and Meta-Analyses (PRISMA) guidelines guided all methodology.

**Results:**

Thirteen intervention trials were identified that met our inclusion criteria and measured change in HED. Studies were judged of moderate quality. A beneficial effect of non-pharmacological interventions on HED was reported in six studies, three of which were deemed clinically significant by the review authors; no statistically significant effects were identified in the other seven studies. Interventions achieving statistical and/or clinical significance had an intervention dose of two hours or greater, used an array of psychosocial approaches, including Motivational Interviewing integrated in Brief Intervention, cognitive behavioral therapy and integrated risk reduction interventions, and were delivered both individually and in groups.

**Conclusions:**

Evidence for the effectiveness of non-pharmacological interventions to reduce HED in sub-Saharan African settings was limited, demonstrating the need for more research. To strengthen the literature, future research should employ more rigorous study designs, improve consistency of HED measurement, test interventions developed specifically to address HED, and explore structural approaches to HED reduction.

## Introduction

Alcohol is a widely used psychoactive substance, with 47% of individuals over the age of 15 reporting alcohol use in the past year, globally [1]. Alcohol consumption is associated with a myriad of poor health outcomes for the individual consuming alcohol, as well as for others around them (e.g. driving while intoxicated, violence while under the influence). In 2016, the Global Burden of Disease Collaboration identified alcohol as the seventh leading risk factor for death, globally [2]. The Global Burden of Disease 2017 study further found that among young persons aged 15–49 years, alcohol use is the leading risk factor for premature death and burden of disease, which has been the case since 1990 [3]. More recently, a comparative risk assessment has affirmed alcohol use continues to be a leading risk factor for death [4]. The impact of alcohol consumption on health outcomes is often dependent on two factors: the total volume of alcohol consumed and the pattern of alcohol use [5]. Harmful alcohol use is defined by the World Health Organization (WHO) as “drinking that causes detrimental health and social consequences for the drinker, the people around the drinker and society at large, as well as patterns of drinking that are associated with increased risk of adverse health outcomes” [6]. Harmful alcohol use is associated with increased risk of morbidity and mortality and has been causally linked to hundreds of diseases and injuries [2, 5, 7]. Globally, patterns of alcohol use, the volume of alcohol per capita consumed (APC), and the subsequent health and social consequences vary greatly by region and country.

The WHO Africa Region has an APC of 6.3 liters per person, which is comparable to the global average of 6.4 liters per person. However, this is attributed to a large proportion of the population abstaining from drinking altogether, especially women [5]. Among men and women (15 years of age or older) who do use alcohol, total APC is high at 18.4 liters per person (compared to 15.1 liters among alcohol users, globally), suggesting that those who drink alcohol in Africa consume more than drinkers in other parts of the world [5]. One pattern of harmful alcohol use that is particularly hazardous to health is heavy episodic drinking (HED), also known as binge drinking, although the two have slightly different definitions.

The WHO defines HED as consuming at least 60 grams of pure alcohol on at least one occasion in the past 30 days [5]. The U.S. National Institute on Alcohol Abuse and Alcoholism (NIAAA) defines binge drinking as drinking until one’s blood alcohol level exceeds 0.08 g/dL, which is typically achieved by four drinks within two hours for women or five drinks within two hours for men (based on the United States’ definition of a standard drink, i.e., 14 grams of pure alcohol) [8]. HED is prevalent in sub-Saharan Africa with over half of drinkers (50.2%) in the WHO Africa Region engaging in this behavior (compared to 39.5% of drinkers globally) [5]. While there has been a slight decline in the prevalence of HED among drinkers in the Africa Region since 2000 (from 55.5% to 50.2%), sub-Saharan Africa continues to experience the highest prevalence of HED among alcohol users globally [5]. In the Africa Region, HED is most prevalent among persons aged 20 to 24 years (50.7% among drinkers) and men are more than twice as likely to engage in HED than women (60.5% of drinkers compared to 28.2%) [5]. Prevalence of HED varies substantially throughout sub-Saharan Africa. In the Republic of Congo, Gabon and Equatorial Guinea, prevalence among drinkers exceeds 80%, while prevalence in Niger, Senegal, Chad and Guinea hovers just below 35% [5].

HED has been linked to increased risk of injury and cardiovascular disease [5, 9], and harmful alcohol consumption generally in sub-Saharan Africa is associated with an array of health and social problems, including infectious diseases [5, 9, 10]. Given the heavy burden of both HIV and tuberculosis (TB) in sub-Saharan Africa, HED is of special concern in the region [11, 12]. A meta-analysis of experimental studies found a dose response relationship between alcohol use and intention to engage in condomless sex, which increases risk of HIV acquisition [13]. Another meta-analysis found that individuals that drink heavily had a three-fold greater risk of TB infection (pooled relative risk 2.94, 95% CI: 1.89–4.59) [14]. Heavy alcohol use is also associated with poor engagement and retention in HIV and TB care, as well as accelerated disease progression, which is especially problematic in a generalized HIV epidemic; adherence to treatment not only improves health outcomes but also reduces the risk of further transmission [14–16]. Similarly, poor adherence to TB treatment can lead to complications, such as the development of drug resistance.

Pharmacological interventions involve the use of pharmacotherapy in the treatment of alcohol misuse. Pharmaceuticals can be used to aid in the withdrawal process (e.g. benzodiazepines, phenobarbital, anticonvulsants) among persons who are physically dependent as well as to promote abstinence (e.g. naltrexone, disulfiram) [17]. They can be used in conjunction with non-pharmacological approaches or as a stand-alone intervention. Despite the pervasiveness of HED in sub-Saharan Africa, there are limited resources available to address this health issue, including limited availability of pharmacological alcohol treatment options [18]. When available, medications (especially newer ones such as naltrexone) tend to be expensive and many individuals lack health insurance to subsidize costs [17]. Furthermore, for those that do have access to insurance, treatment of alcohol use disorders is often not covered, making finances a significant barrier to pharmacological treatment access in low income countries [19].

Non-pharmacological interventions can involve the use of psychosocial and structural approaches to address alcohol misuse but they do not include a pharmaceutical/medication component. Psychosocial interventions are defined as “psychologically-based interventions aimed at reducing consumption behavior or alcohol-related problems” [20], while structural interventions go beyond the individual level to change the environments in which risk behavior occurs, such as alcohol regulation. Non-pharmacological interventions to reduce harmful alcohol consumption have been piloted and implemented in numerous settings throughout sub-Saharan Africa, but this evidence has not been systematically reviewed and synthesized. Understanding the effect of non-pharmacological interventions on HED is particularly important in the context of sub-Saharan Africa, given elevated rates of this pattern of drinking, and its harmful effects on health.

In order to address this gap, we reviewed the existing literature for non-pharmacological interventions to address alcohol use in sub-Saharan African settings that reported HED outcomes. This review is part of a companion review and meta-analysis [21] with the same search criteria that looked at different alcohol use outcomes (e.g. Alcohol Use Disorders Identification Test [AUDIT] score). We report HED outcomes in the present manuscript separate from the larger meta-analysis [21] as they could not be quantitatively pooled due to heterogeneity in their measurement.

## Methods

This systematic review was guided by both the Cochrane Handbook recommendations for the review of interventions [22] and the Preferred Reporting Items for Systematic Reviews and Meta-Analyses (PRISMA) guidelines (see “S1 Appendix”) [23]. This review was registered with the PROSPERO online registry (registration number CRD42019094509).

In this systematic review, we searched Embase, Medline, PsycINFO, EBSCO, CINAHL, and Cochrane CENTRAL on December 21, 2017 for published reports in English from the earliest available date per database. This search was rerun on March 14, 2019 and again on August 19, 2020. The search protocol is provided in “S2 Appendix”. We also hand-searched reports and included supplementary data sent by study authors. As discussed, this review was part of the search for a larger systematic review inclusive of other alcohol consumption outcomes. The parameters of the search were as follows: randomized or nonrandomized controlled trial design, conducted in sub-Saharan Africa, assessing a non-pharmacological intervention aimed at alcohol reduction, and measuring at least one alcohol consumption outcome.

For this paper, eligible alcohol consumption outcomes were those measuring HED outcomes. Given the wide variability of measurement of these outcomes, we included studies using variations of established measures of HED (e.g., at least 60 grams of pure alcohol on at least one occasion in the past 30 days) and binge drinking (e.g., four drinks for women or five drinks for men on a given occasion). In addition, we included measures indicative of this pattern of drinking (i.e., high consumption of alcohol on at least one occasion), such as high elevated Blood Alcohol Concentration (BAC) or frequency of getting drunk. We determined whether outcomes met these criteria by discussion and consensus among study authors.

Reasons for exclusion included: alcohol reduction not being a primary goal of the intervention; alcohol reduction only being addressed in the context of sex; not having a comparator group; or having a comparator that was another evidence-based or ‘bona-fide’ alcohol intervention (i.e., non-inferiority trial) as the aims and effect size would differ from that of an efficacy/effectiveness trial.

Eligible comparator groups included interventions unrelated to alcohol, usual care for alcohol or other services, brief feedback on an alcohol screening tool, alcohol or other informational materials, wait-list, and nothing.

One author (KS) screened all titles and abstracts. A second author (AM) did a targeted review of the screened titles and abstracts. All authors and three research assistants reviewed full-text reports and assessed their eligibility for inclusion in pairs. The standardized rubric that was used for the review of full-text articles is provided in “S3 Appendix.” Disagreements between pairs of reviewers were resolved by discussion and consensus was reached between the reviewers, or by a third author.

### Data extraction and quality assessment

Three authors (AM, KS, TH) independently extracted all outcome data into standardized, piloted data collection forms (“S4 Appendix” includes the data extraction form and all extracted data). Population characteristics, as well as characteristics of the study design, intervention, and comparator of each study were extracted by one of the reviewers and checked by the second reviewer for accuracy. All data related to the study’s primary findings specific to intervention effect were independently extracted by both reviewers, compared, and reconciled through discussion. Corresponding authors of included studies were contacted to collect relevant data not reported in the paper.

Study quality was assessed at the study-level using the Cochrane Collaboration Tool for Assessing Risk of Bias (see “S5 Appendix”) [22]. The Cochrane Collaboration’s recommended approach requires the review and assessment of each study under the follow types of bias: (1) Selection bias (sequence generation and allocation concealment); (2) Performance bias (blinding of participants and providers); (3) Detection bias (blinding of outcome assessors); (4) Attrition bias (incomplete outcome data); (5) Reporting bias (selective outcome reporting). One additional source of bias were assessed following the GRADE handbook [24] given the review’s inclusion of quasi-experimental controlled trials: (6) failure to adequately control for confounders. Assessment of risk of bias occurred at the time of data extraction. Pairs of reviewers (AM, KS, TH) independently rated each of the items as low risk, high risk, or unclear. Discrepancies were resolved by discussion. If consensus could not be reached, a third author was asked to break the tie. All studies were included in the review regardless of risk of bias (per our review protocol).

### Data analysis

The disparate measurement of HED outcomes did not allow for the pooling of study findings through meta-analysis. Therefore, we summarize each study and report quantitative findings for studies individually. We report effects in the format that they were reported in the original paper by study authors. In addition, the authors assessed the clinical significance of the studies’ findings through discussion and consensus. The decision was made based on effect size or change in the amount of HED pre- and post-intervention, while taking into account other available information (e.g., population, setting, baseline drinking) [25]. Since interventions to reduce HED, especially Brief Intervention (BI), commonly aim to reduce immediate risk, short-term change (e.g., 3 months) and change not sustained over longer time periods still had the potential to be judged as clinically meaningful [26].

### Role of the funding source

Sponsors of the study authors had no role in study design, data collection, data analysis, data interpretation, or writing of the report. The authors had full access to all data and the final responsibility for the decision to submit for publication.

## Results

**Fig 1:**
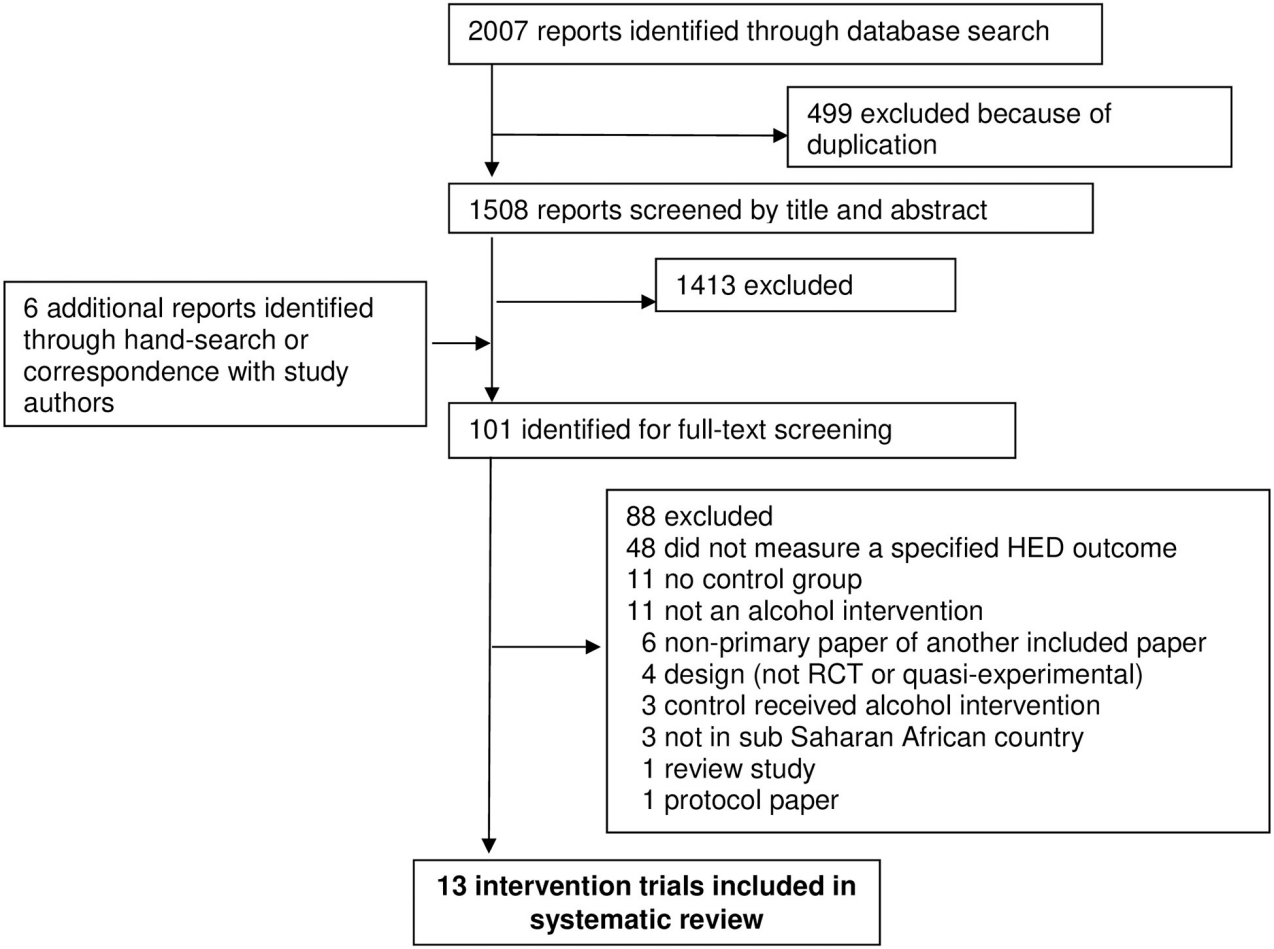
Studies included in systematic review. Adapted from the 2009 PRISMA Flow Diagram. Moher D, Liberati A, Tetzlaff J, Altman DG, The PRISMA Group. Preferred Reporting Items for Systematic Reviews and Meta Analyses: The PRISMA Statement. *PLoS Med* 2009;6(7): e1000097. doi:10.1371/journal.pmed1000097.

A total of 1508 unique citations were identified through the database search after the exclusion of duplicates. Six additional studies were identified through hand-searching and correspondence with study authors. Of the 101 reports that underwent full-text screening, 88 were excluded for reasons outlined in Fig 1. See “S6 Appendix” for a list of ineligible studies reviewed as full-text with reasons for exclusion. In total, 13 studies met criteria for inclusion in this review [27–39].

### Characteristics of included studies

Characteristics of the study design and samples for each included study are summarized in Table 1. The thirteen studies included in this review were conducted in four countries: South Africa (n = 9) [27, 31, 33–39], Kenya (n = 2) [30, 32], Nigeria (n = 1) [29], and Zimbabwe (n = 1) [28]. Studies included randomized control trials (n = 6) [30–32, 35–37], cluster randomized control trials (n = 5), [27, 28, 34, 38, 39], one non-randomized controlled trial [29], and one quasi-experimental pretest/posttest design [33]. The majority of studies included general adult populations, whereas one was with university students [36], two with young adults [31], and one with women 15 years or older [39]. Three studies included patient populations, including general outpatients [35], HIV outpatients [32], and TB patients [34]. Two workplace-based studies were identified, one with safety and security employees [27] and another with employees of alcohol establishments [33]. Another study included market traders [29]. One study was with female sex workers [30], and two were focused on fetal alcohol syndrome prevention among pregnant women [38] and women at-risk for alcohol-exposed pregnancy [37]. Of the thirteen studies included, seven included alcohol misuse at baseline as part of their eligibility criteria [30–32, 34–37].

**Table 1:** Summary of study and population characteristics of non-pharmacological alcohol interventions in sub-Saharan Africa.

Author, year	Country	Data collection years	Study design	Population (alcohol use eligibility criteria at baseline)*	Total N*	Age (SD)*	% female*
Burnhams, 2015 [27]	South Africa	2011–2012	CRCT	Safety and security employees	325	41.7 (NR)	13.0%
Cubbins, 2012 [28]	Zimbabwe	2003–2007	CRCT	Adults (18–30)	5,543	21.8 (3.3)	47.0%
Eze, 2020 [29]	Nigeria	2016	NRCT	Market traders	376	42.16 (NR)	43.0%
L’Engle, 2014 [30]	Kenya	2011–2012	RCT	Female Sex Workers (AUDIT = 7–19)	818	27.5 (6.6)	100.0%
Mertens, 2014 [31]	South Africa	2008	RCT	Young adults (18–24) (binge drinking 5 drinks+ for men, 3 drinks+ women, or any illicit drug use in the prior year)	403	21 (NR)	52.0%
Papas, 2020 [32]	Kenya	2012–2016	RCT	HIV-infected outpatients (AUDIT-C = 3 or 6 or more drinks per occasion at least monthly)	614	38.9 (8.0)	51.5%
Peltzer, 2006 [33]	South Africa	NR	Quasi-Experimental Pretest/Posttest	Employee sector of licensed establishments	18 managers & servers (received intervention); 309 patrons (BAC assessed)	NR	NR
Peltzer, 2013 [34]	South Africa	2011–2012	CRCT	Tuberculosis outpatients (AUDIT ≥ 8 for men; AUDIT ≥ 7 for women)	1,196	36.7 (10.9)	25.7%
Pengpid, 2013 [35]	South Africa	2011–2012	RCT	Outpatients (AUDIT = 8–19 for men; AUDIT 7–19 for women)	392	35.6 (NR)	27.0
Pengpid, 2013 [36]	South Africa	2011–2012	RCT	University students (AUDIT > 8)	152	21.9 (3.5)	12.7%
Rendall-Mkosi, 2013 [37]	South Africa	2007–2008	RCT	Women at high risk for alcohol effected pregnancy	165	29.8 (NR)	100.0%
Rotheram-Borus, 2019 [38]	South Africa	2009–2016	CRCT	Pregnant women	1,236	26.4 (NA)	100.0%
Wechsberg, 2019 [39]	South Africa	2012–2014	CRCT	Black African women (15 or older)	641	29.8 (7.8)	100.0%

Notes:

* indicates as reported at baseline;

RCT = randomized controlled trial; CRCT = cluster randomized controlled trial; NR = not reported; AUDIT = Alcohol Use Disorders Test; All studies were peer-reviewed; Papas, 2020 did not report on HED but emailed data to study authors.

### Description and effects of interventions

A description of the included interventions, comparator groups, and the reported intervention effects on HED outcomes are reported in Table 2. Table 2 also includes outcome definitions, as measured by each study. Only one study included a biological outcome, which was BAC [33]; all other studies included self-reported drinking outcomes.

**Table 2:** Intervention details and summary of the intervention effect on reduction in heavy episodic drinking (HED) outcomes.

Author, year	Population, Country	Intervention description	Comparator description	Outcome definitions	Summary of results of the intervention effect on HED outcomes
Burnhams, 2015 [27]	Safety and security employees, South Africa	The intervention was Team Awareness, a workplace training program addressing behavioral risks among municipal employees. It consisted of 6 training modules delivered in group sessions by local interventionists in a municipality facility over a total of 8-hours (4-hour sessions over 2 weeks). The aim was to reduce risky drinking, alcohol-related HIV risk, and increase help-seeking behavior.	One hour wellness session	Binge drinking defined as ≥ 5 drinks at one sitting in the prior 30 days	Significant intervention effect for reduction in the mean number of days having five or more drinks in one sitting at 3 month follow-up.
Cubbins, 2012 [28]	Adults (18–30), Zimbabwe	Based on the theory of diffusion of innovations, the Community Popular Opinion Leader intervention used popular opinion leaders to spread culturally-specific health messages on reducing HIV-related risk and not drinking excessively. ~60 individuals per sample site were recruited from the social networks of patrons of micro-venues (e.g., bottle stores or general dealers), attending a 2-week training on messages plus refresher trainings.	No comparator intervention	Frequency of getting drunk defined as the number of days respondent reported getting drunk in the prior 30 days	No support for an intervention effect on frequency of getting drunk at the community-level or individual-level at 12- or 24-month follow-up. No gender effects found.
Eze, 2020 [29]	Market traders, Nigeria	A group lifestyle/behavioral modification program consisting of two 5-hour sessions of on-site health education on prevention, early detection and control of hypertension through increased physical activity and dietary adjustment delivered by a public health physician, a dietician and a physical fitness counsellor. Sessions included lectures, dietary and exercise demonstrations, educational materials, diary tracking, and weekly SMS on risk reduction based on the Health belief model. Outreach health posts were established to provide re-enforcement of the intervention.	No comparator intervention	Excessive alcohol consumption was defined as 5 standard drinks/day if male, and 4 standard drinks if female on 5 days in the prior 30 days	Significant intervention effect on reduction of alcohol consumption in intervention compared to control at 6-month follow-up.
L’Engle, 2014 [30]	Female Sex Workers engaged in hazardous /harmful drinking, Kenya	The intervention was based on the WHO’s BI for Alcohol Use [40], used motivational interviewing techniques, and contained elements from stages of change and social cognitive health behavior change theories. Nurse counselors provided the intervention over six 20-minute one-on-one sessions in a health facility. The goal was to reduce alcohol consumption among female sex workers.	Nutrition comparator intervention	Frequency of binge drinking defined as ≥ 3 drinks on same occasion in the prior 30 days; measured on a 4-point Likert scale (“Never” to “Most of the time”)	Significant intervention effect on reduction in frequency of binge drinking in intervention compared to control at 6-month and 12-month follow-up.
Mertens, 2014 [31]	Young adults (18–24) engaged in binge drinking or illicit drug use, South Africa	The intervention was a nurse-delivered single-session (total time not reported) brief motivational intervention, provided with a resource list for drinking and drug use problems. It was delivered in a public health clinic to young adults who screened for heavy alcohol or illicit drug use. Primary Care Nurse Practitioners were trained in brief motivational intervention for alcohol and drug misuse.	Usual care + resource list	Heavy drinking defined as ≥ 5 drinks if men and ≥ 3 drinks if women on a single occasion (1 drink = 12 g alcohol)	No significant differences in heavy drinking between intervention and control at 3-month follow-up.
Papas, 2020 [32]	People living with HIV engaged in hazardous /harmful drinking, Kenya	The Kenya Health Behavior Study delivered cognitive-behavioral therapy (CBT) over 6 weekly gender-segregated group sessions (90-minutes each) delivered by a counselor in a health facility setting. The goal was alcohol abstinence for HIV outpatients.	Group healthy lifestyles education comparator intervention	Heavy drinking days defined as ≥ 4 standard drinks if male, and ≥ 5 standard drinks if female (converted to US standard 14g) in the prior 60 days	Significant intervention effect for reduction in the number of heavy drinking days in the prior 6 months at 7–30 weeks follow-up, but not maintained at 31–46 weeks.
Peltzer, 2006 [33]	Servers and mangers of alcohol establishments received intervention; BAC tested on bar patrons, South Africa	The intervention was a training program on the prevention of intoxication and related problems of bar patrons for managers and servers of alcohol establishments. A 5-hour program was delivered for servers and a 6-hour program for managers. Curriculum aimed to increase relevant knowledge (e.g., alcohol laws, signs of intoxication); behavioral skills (e.g., estimating BAC by drink counting); communication methods; and policy recommendations.	No comparator intervention	Breathalyzer test for BAC level of >1.0% among bar patrons at training sites	No support for an intervention effect on overall BAC at 3-month follow-up. Tests of significance not conducted.
Peltzer, 2013 [34]	Tuberculosis outpatients engaged in hazardous /harmful drinking, South Africa	The intervention was based on the WHO BI for Alcohol Use [40], with additional content informed by Information-Motivation-Behavioral Skills Model [41]. Two one-on-one sessions were delivered (15–20 minutes each) by a lay counselor in a health facility within one month.	Health education leaflet on responsible drinking	Frequency of HED defined as ≥ 5 standard drinks on one occasion if male, and ≥ 4 standard drinks if female (1 drink = 12 g alcohol)	The intervention effect was not statistically significant for HED at the 6-month follow-up; there were significant reductions in HED over time in both intervention and control groups.
Pengpid, 2013a [35]	Outpatients engaged in hazardous /harmful drinking, South Africa	The intervention was based on the WHO BI for Alcohol Use [40], with additional content informed by Information-Motivation-Behavioral Skills Model [41]. A single session was delivered (20 minutes) by a research assistant in a hospital, including personalized feedback on AUDIT results, a health education leaflet, simple advice plus brief counselling to reduce excessive drinking.	Health education leaflet on responsible drinking	Frequency of HED defined as ≥ 5 standard drinks on one occasion if male, and ≥ 4 standard drinks if female	The intervention effect was not statistically significant for HED at the 12-month follow-up; there were significant reductions in HED over time in both intervention and control groups.
Pengpid, 2013b [36]	University students engaged in hazardous /harmful drinking, South Africa	The intervention was based on the WHO BI for Alcohol Use [40], with additional content informed by Information-Motivation-Behavioral Skills Model [41]. A single session was delivered (20 minutes) by a nurse research assistant in a public venue, including feedback on AUDIT results, a health education leaflet, simple advice plus brief counselling to reduce excessive drinking.	Feedback on AUDIT + health education leaflet on responsible drinking	HED defined as ≥ 5 standard drinks on one occasion if male, and ≥ 4 standard drinks if female (1 drink = 12 g alcohol)	Significant intervention effect; overall reduction in HED in both intervention and control arms, with a significantly higher decline in the intervention compared to control at 6- and 12-month follow-up.
Rendall-Mkosi, 2013 [37]	Women at high risk for alcohol effected pregnancy, South Africa	The intervention included 5 motivational interview sessions, plus an informational pamphlet on fetal alcohol syndrome prevention and a handbook on woman’s health. The sessions were delivered one-on-one by lay counselors over 2-months at a location convenient to women. The aim was to increase contraceptive use and reduce risky alcohol use among women of reproductive age at risk for alcohol-exposed pregnancy.	Informational pamphlet on fetal alcohol syndrome prevention + women’s health handbook	Risky drinking defined as > 5 drinks at one sitting in past 3 months, or > 7 drinks in a week	The intervention effect was not statistically significant for at-risk drinking at 3-months or 12-months follow-up. There were declines for both groups in the proportion of participants who met the criteria for risky drinking at 3- and 12-month follow-up compared to baseline.
Rotheram-Borus, 2019 [38]	Pregnant women, South Africa	The “Philani Program” trained women from the community to provide home visitation to pregnant women as “Mentor Mothers.” Training included cognitive-behavioral change strategies and maternal health education (i.e., HIV/TB prevention, PMTCT, problematic alcohol use, breastfeeding, nutrition). They were trained to provide 1 brief alcohol intervention specific to fetal alcohol syndrome prevention. At least 4 antenatal and 4 postnatal visits were provided within 2-months of childbirth.	Standard-of-care maternal and child health and PMTCT services	Problem drinking defined as ≥ four 14-g glasses in one day at least once a month, and at least one symptom of alcohol withdrawal on the AUDIT-C	The coefficients showed that drinking increases over time, whereas the intervention attenuated this. However, this intervention effect only became substantial at the 5-year time point. Tests of statistical significance not conducted.
Wechsberg, 2019 [39]	Black South African women (15 or older), South Africa	Women in the “Women’s Health CoOp Plus” arm underwent HIV counseling and testing and participated in 2 one-on-one intervention sessions (1 hour each) 1 week apart. Sessions took place at the study site facilitated by an interventionist from the community. The sessions aimed to educate participants about the risks of alcohol and other drug use, including how alcohol and drug use and sexual risk are related to HIV for women and gender power. Sessions covered risk-reduction strategies and included role-play and rehearsal.	Standard-of-care HIV counseling and testing	Frequent heavy drinking defined as heavy drinking (4 or more drinks) on 11 or more days in the past 30 days	Significant intervention effect at 6-month follow-up on reduced frequent heavy drinking and fewer heavy drinking days, but not maintained at 12-months follow-up.

Notes: HED = heavy episodic drinking; WHO = World Health Organization; BI = Brief Intervention; BAC = Blood Alcohol Concentration; PMTCT = preventing mother to child transmission of HIV; Standard drink size definition not reported in all studies. In South Africa, 1 standard drink = 12 g alcohol; Papas, 2020 did not report on HED but emailed data to study authors.

Interventions were primarily individual-level psychosocial interventions, including seven utilizing Motivational Interviewing (MI) and/or Brief Intervention (BI) in single or multi-sessions [30, 31, 34–38]. Other psychosocial intervention approaches identified include: three multi-component risk reduction interventions, including two alcohol/HIV risk reduction interventions [27, 39] and one focused on risk factors for hypertension [29], as well as one cognitive-behavioral therapy (CBT) intervention [32]. Two studies were identified that targeted change beyond the individual-level. At the interpersonal and community-levels, one intervention employed a social-network intervention approach based on the Diffusion of Innovation Theory [28], and another organizational-level intervention provided training to employees of alcohol establishments in the responsible sale and serving of alcohol [33]. The details of the intervention effects are presented in the next section organized by intervention approach.

Comparator groups most commonly included an educational leaflet or general information (n = 4) [34–37], standard-of-care health services (n = 3) [31, 38, 39], or nutrition or lifestyle interventions (n = 3) [27, 30]. Three studies did not provide any treatment to the comparator group [28, 29, 33].

In general, results from six of the thirteen trials showed at least one statistically significant change in a HED outcome in the expected direction among intervention compared to those in comparator groups. Only three of these studies were deemed clinically significant by review authors. The other seven trials reported no intervention effect of HED at a level of statistical significance.

#### Description of interventions and intervention effects on HED outcomes

*Individually-based Motivational-Interviewing (MI)*, *Brief Interventions (BI)*. Eleven out of the thirteen interventions identified in this review were individual-level psychosocial interventions. Of these interventions, the most commonly used intervention approach was MI interventions, which was the focus of seven interventions [27, 30–34, 36], six of which were described as BI [27, 30–32, 34, 36] with four based on the WHO BI for Alcohol Use [27, 31, 32, 37]. Despite commonalities in the core approach, implementation across studies varied. The total intervention dose ranged from single 20-minute sessions to a 120-minute 6-session intervention. MI/BI interventions were most commonly held in health facilities, but also took place in participants’ homes and other community venues. Only two out of the seven MI/BI studies reported statistically significant changes in one of the review’s specified HED outcomes.

One of the two interventions reporting statistically significant results was L’Engle et al.’s [30] study with Kenyan female sex workers, which included six 20-minute individually-delivered BI sessions using MI to reduce alcohol use with dual focus on HIV risk reduction. Those receiving the intervention reported drinking 3 or more drinks on the same occasion less frequently in the prior month at 6 months compared to participants receiving a time-matched nutrition intervention (Adjusted Odds Ratio [AOR] = 0.13, 90% Confidence Interval [CI] = 0.10, 0.17, *p* < 0.0001) and 12 months follow-up (AOR = 0.18, 90% CI = 0.13, 0.23, *p* < 0.0001). The review authors deemed these findings clinically significant, given the effect size and sustained change. The second was Pengpid et al.’s [36] study focused on South African university students who drink at hazardous levels. The intervention was based on the WHO BI for Alcohol Use [38], with additional content informed by the Information-Motivation-Behavioral (IMB) Skills Model [39]. Similar to L’Engle et al. [30], the intervention was delivered individually; however, it included only a single 20 minute session as opposed to six. Pengpid et al.’s [36] reported statistically significant reductions in HED (≥ 5 standard drinks on one occasion if male and ≥ 4 standard drinks if female) over time across both treatment groups. The respondents who received BI showed a higher decline in HED during the follow-up compared to control participants at the 12-month follow-up (β = −0.44; 95% CI = −0.76, −0.12; *p* = 0.007); however, these small changes were not judged as clinically significant by the review authors.

The other five interventions incorporating MI did not report statistically significant reductions in the review’s alcohol outcomes [31, 34, 35, 38, 41]. Three of these studies employed one to two (15–20 minute) BI sessions with patient populations in clinic settings in South Africa, including young adults in primary care [27], TB patients [34] and hospital outpatients [35]. Two of the trials included the same intervention reported in Pengpid et al. [36] (WHO BI for Alcohol Use with IMB model modifications) using a health education leaflet as control [34, 35]. In both trials, reductions in HED (i.e., drinking ≥ 5 standard drinks for men and ≥ 4 standard drinks for women on one occasion) were observed in both intervention and control. However, unlike Pengpid et al., [32] there was not a statistically significant difference between intervention and control.

The two remaining MI interventions had commonalities in their goals to reduce drinking during pregnancy among South African women [37, 38]. Rotheram-Borus et al. [38] reported on a single-session alcohol BI integrated into a home visiting intervention delivered by locally trained “Mentor Mothers” aimed at fetal alcohol syndrome prevention in South Africa. Rendall-Mkosi and colleagues [37] tested the effectiveness of a five-session, individually-based MI intervention to reduce the risk of alcohol exposed pregnancy among South African women of reproductive age screened as at-risk for alcohol exposed pregnancy. Both saw trends towards improvement in HED measures. However, Rendall-Mkosi and colleagues [34] did not reach statistical significance. Rotheram-Borus et al. [30] reported drinking increased over time in both study arms, and that the intervention attenuated an increase in problem drinking. The study authors report that the intervention effect became “substantial” at the 5-year time point for problem drinkers, with the proportion of mothers classified as problem drinkers approximately 6% in the intervention arm and 12% in the control arm. Despite this difference, we classify this study with other studies that show no statistical significance, as the authors did not explicitly test the statistical significance of this effect.

*Integrated risk reduction interventions*. Three interventions, including two group and one individually-based, included a risk reduction approach focused on alcohol within a broader multi-component intervention package, including two focused on HIV [27, 39] and one on risk factors for hypertension [29]. All three reported statistically significant reductions in HED outcomes. Among the alcohol/HIV risk reduction interventions was “Team Awareness” and “Women’s Health CoOP Plus” (WHC+) [27, 39]. Employees receiving the Team Awareness intervention reported a statistically significant reduction in frequency of binge drinking in the prior 30 days [*F*(1,117) = 25.16, *p* <0.0001)]. The mean number of days participants in the intervention condition reported having five or more drinks in one sitting in the prior 30 days reduced from 2.1 days to 1.4 days, in the predicted direction. The review authors did not deem this change clinically significant; drinking five or more drinks on at least one day in the past month still fits within the definition of binge drinking. WHC+ was delivered over two 1-hour one-on-one sessions aimed to reduce alcohol and other drug use among women living with HIV in Cape Town. WHC+ participants were significantly less likely to engage in frequent heavy drinking at 6 months follow-up (4 or more drinks on 11 or more days in the prior 30 days) (AOR = 0.45; 95% CI = 0.28, 0.73; p = 0.02), but not at 12-month follow-up (AOR = 0.71, 95% CI = 0.43, 1.18; p = 0.19). Participants in the WHC+ also reported less days of heavy drinking (4 or more drinks) in the prior 30 days at 6-month follow-up (*p* = 0.01), but not 12-month follow-up (*p* = 0.36). The review author deemed the reduction in the proportion of women engaging in frequent heavy drinking as clinically significant, reducing from nearly 40% to 20% at 12-months follow-up. While there was also considerable change in the control condition, we still deemed the difference meaningful.

Eze et al. [29]’s risk reduction intervention focused on the reduction of alcohol use as a risk factor of hypertension, while simultaneously aiming to reduce other risk factors (e.g., poor diet, physical inactivity). The intervention included two 5-hour group sessions with market traders in Nigeria, as well as the establishment of health posts in the region for reinforcement of intervention content and blood pressure screening. Compared to the control, intervention participants saw a statistically significant reduction in binge/excessive drinking (5 standard drinks/day if male, and 4 standard drinks if female on 5 days in the prior 30 days) (χ^2^ = 15.09, p <0.001). This change was considered clinically significant by the review authors, with the proportion of binge alcohol drinkers reduced by 9.5% among the intervention group.

*Cognitive behavioral therapy*. One study employed group-delivered cognitive behavior therapy (CBT). The Kenya Health Behavior Study assessed the efficacy of a six-session gender stratified group cognitive behavioral therapy (CBT) intervention delivered by counselors to reduce alcohol use among HIV outpatients in Kenya [32]. Compared to a healthy lifestyle control, intervention participants reported statistically significant reductions in the number of heavy drinking days (≥ 4 standard drinks if male and ≥ 5 standard drinks if female) in the prior 60 days at 7–30 weeks follow-up (mean difference [MD] = -0.21; 95% CI = -0.35, -0.08; *p* = 0.002). However, these effects were not maintained at the 31–46 week follow-up (MD = -0.02; 95% CI = -0.09, 0.06; *p* = 0.67). The average change in heavy drinking days from baseline to 7–30 weeks follow-up changed from approximately 6 days on average to 0 days, which the review authors deemed a clinically meaningful change. Although this was a statistically significant change compared to control, similarly meaningful change was observed in the control group. The review authors did not view this difference between intervention and control as clinically significant.

*Interventions targeted change beyond the individual-level*. Two studies, Cubbins et al. [28] and Peltzer et al. [33], implemented interventions at a community or organizational-level, but did not report any statistically significant change in alcohol outcomes. Cubbin et al.’s [28] community approach was based on Diffusion of Innovation Theory, employing a popular opinion leader model to spread culturally-specific health related messages across social networks in rural Zimbabwe. The intervention was targeted at the individual level but was expected to diffuse throughout a community via word of mouth and social normative influence. However, no statistically significant differences were found between communities receiving the CPOL intervention compared to those that did not in the number of days respondents reported getting drunk in the prior 30 days at either time point, nor was there significant individual-level change in this outcome. Peltzer et al.’s [33] structural intervention targeted change at the organizational-level, conducting a quasi-experimental controlled trial to assess the effects of a responsible alcohol beverage sales and servicing training intervention with owners and servers from alcohol serving establishments in Gugulethu, South Africa. A cross-sectional design was employed to assess pre/post changes of BAC level of bar patrons. Although tests of statistical significance were not reported, changes in the intervention sites and the control sites were not in the expected direction.

### Risk of bias

**Fig 2:**
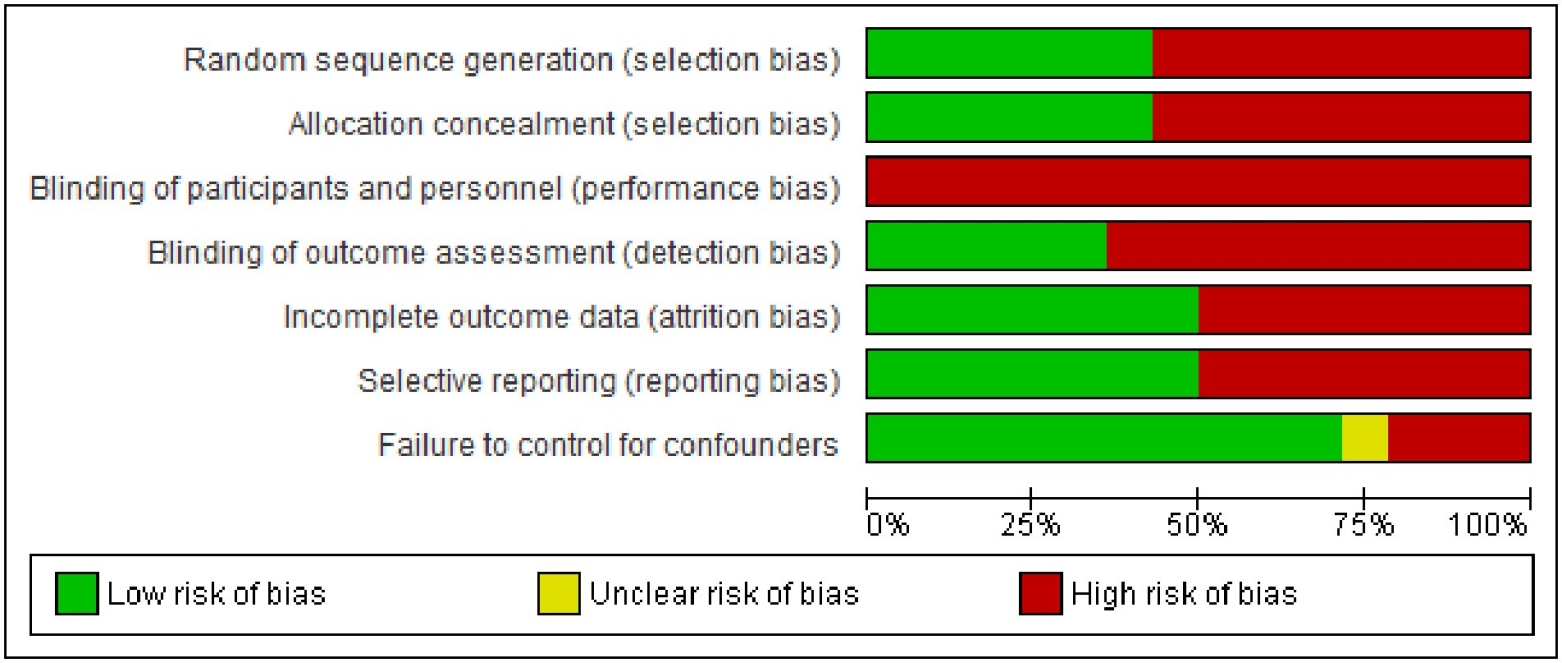
Risk of bias graph: Review authors’ judgements about each risk of bias item presented as percentages across included studies.

In general, included studies evaluated with the Cochrane risk of bias tool were of moderate quality (see Fig 2). Just under half of studies were judged as low risk for random sequence generation [30, 32, 34, 35, 37, 39] and allocation concealment [30–32, 34, 35, 37], with the other half judged as high risk. None of the studies blinded both participants and study personnel, and only one third of studies blinded outcome assessment [29, 30, 34–36]–potential sources of performance and detection bias. Other methodological weaknesses included a lack of published study protocols resulting in high risk for selective reporting bias in half of studies [29–31, 33, 37, 39], and attrition bias due to loss to follow-up being greater than 20% [27, 33–35, 37]. Just under 25% of studies were judged as high-risk for failure to control for potential confounders [33, 37, 38]. The full risk of bias assessment per study is included in “S7 Appendix” and a graphic depiction of the assessment results per study is provided in “S8 Appendix.”

## Discussion

This systematic review of non-pharmacological interventions aimed to reduce alcohol consumption in sub-Saharan Africa identified 13 trials that measured change in HED outcomes. Although sub-Saharan Africa has among the highest occurrence of HED in the world and high rates of alcohol-related morbidity and mortality, this is the only review to synthesize the effect of non-pharmacological alcohol interventions on HED outcomes in sub-Saharan Africa to-date. The majority of studies evaluated individual-level psychosocial interventions, such as BI with MI and other individual or group psychosocial approaches. Six of the trials showed statistically significant reductions in HED, three of which the review authors judged as clinically significant. Seven of the trials showed no effect. Alcohol interventions achieving statistical and/or clinical significance were conducted across diverse settings and populations, using a range of psychosocial approaches, including MI integrated in BI, CBT, integrated risk reduction interventions, delivered both individually and in groups. Taken together, the picture remains unclear regarding which interventions show the most promise for reducing HED outcomes in sub-Saharan African settings, pointing to the need for more research. However, this review can shed light on approaches for future investigation, as well as gaps in the literature.

The most commonly employed intervention approach included in this study was MI, commonly integrated into single or multi-session BI, employed in seven studies. Despite the wide use of this intervention approach, this review provides limited evidence supporting its effectiveness at reducing HED in sSA, with only two studies showing statistically significant effects, one of which was not considered clinically meaningful. This finding coincides with the companion meta-analysis we conducted as part of this same search; the meta-analysis similarly found limited evidence for MI and the WHO SBI guidelines in changing AUDIT scores in sub-Saharan Africa [21]. However, several of the MI-based studies in this review were trending towards significance or saw an improvement in both treatment arms but no significant differences between intervention participants compared to control. The latter could be an effect of the standard comparator for this intervention approach being minimal alcohol intervention (e.g., feedback on AUDIT screening and an alcohol leaflet), which could drive null effects. Nevertheless, these findings are in contrast to a large body of research in high-income country settings that reports moderate effects at alcohol reduction achieved through MI-based BI [42], warranting further investigation.

Based on a qualitative comparison of interventions reporting statistically significant changes in HED outcomes vs. those that did not, there were no clear patterns in differences in success between individual vs. group format of intervention delivery. However, interventions with greater dosage (2 hours+) tended to be more successful than BIs. This is another potential explanation for the underwhelming effects of MI-based BI studies in this review. While this points researchers towards the use interventions with a greater dose and intensity, the scale-up of more intensive approaches is challenged by time and resource constraints common to resource-limited settings. A cost-benefit analysis associated with the CBT Kenya study [32] reported CBT can be effectively and economically task-shifted to paraprofessionals in Kenya [43]. More costing research like this, as well as hybrid implementation studies aimed to assess implementation and effectiveness, are needed in order to better understand the appropriate intervention dosage that could be feasibly scaled up in sub-Saharan African clinical and community settings.

Of the studies included in this review, the overwhelming majority tested psychologically driven interventions focused on individual-level change, such as through MI or other forms of individual or group counseling and education. Only two studies went beyond an individual-level approach, taking an organizational or community-level approach to alcohol reduction [25, 29]. Despite the lack of support found for these interventions, they represent important attempts to address influences of alcohol use beyond individual-level knowledge, motivation, and self-efficacy. Research has established the importance of higher-level factors on alcohol consumption in sub-Saharan African settings, including social and cultural norms [44], alcohol outlet density [45], alcohol marketing [46], and a lack of alcohol regulation and policy enforcement [47]. More research is needed that tests structural intervention approaches in sub-Saharan African settings, such as policy interventions described by the WHO as “best buys” including increases in taxes on alcoholic beverages, bans and restrictions on alcohol advertising, and reductions in retail alcohol availability through reduced hours of sale [44]. These approaches, although difficult to implement, have the largest potential effect in low and middle-income country settings per disability-adjusted life year (DALY) averted.

Although sub-Saharan Africa has among the highest occurrence of HED in the world, no studies were identified in this review that were solely focused on the reduction of this pattern of drinking. The lack of interventions tailored to explicitly reduce HED may in part explain the lack of effect reported on this outcome. A larger literature exists with this aim in high-income country settings, which has demonstrated effects among mainly psychosocial approaches in reducing HED across settings and populations, particularly with adolescents and college students [48–50]. These interventions include a range of psychologically-based interventions, such as BI, personalized normative feedback, and protective behavioral strategies tailored to the dangers of binge drinking specifically [48]. Given strong social and environmental influences of binge drinking, studies targeting HED in high-income country settings tend to be implemented in places where HED occurs (e.g., fraternities, birthday parties), where widespread social norms need to change (e.g., schools), or situations conducive to a teachable moment related to binge drinking (e.g., emergency rooms) [48]. This again highlights the dearth of social and environmental approaches identified in this review; focusing on changing social relationships and environments where HED occurs may strengthen interventions in sub-Saharan Africa. This approach has been used with success in HIV interventions aimed to reduce alcohol-related HIV risk by altering the relationships, norms, and environment of alcohol venues where people engage in alcohol-related sexual risk, including multilevel approaches such as altering proximity to venues, the physical characteristics of venues, and social norms [51].

### Limitations

The trials included in this review were of varying quality, with a number of potential sources of bias identified through the risk of bias assessment. Weaknesses in randomization and allocation concealment methods increases risk for non-representative samples and the risk of confounding factors skewing intervention effects. Detection bias due to non-blinding is also a concern among the studies in this review, which can skew the evidence towards an exaggerated treatment effect. These and other sources of bias identified should be considered in the interpretation of our findings.

Though meta-analysis was originally planned for this review, the inconsistent operationalization of HED limited our ability to quantitatively synthesize the findings across studies in this review. This also prevented a quantitative investigation into heterogeneity to examine differences in intervention effect by study design, intervention approaches, populations, and study settings.

Definitions in HED across studies differed in the quantity of alcohol consumed, definitions of a standard drink, and the timeframe of consumption. Given the already wide variability in measurement, we opted to include several studies with outcomes indicative of HED, including frequency of getting drunk, but this is a subjective measure with high likelihood for inter-individual variability in perceptions of relative intoxication. More consistent outcome operationalization would improve comparability and strengthen the alcohol intervention literature. However, standardized measures of HED may not be possible across studies with such different populations (e.g., adolescents, HIV patients, TB patients, pregnant women). The studies included in this review tended to adapt their outcome definitions to match their study population. In addition, all studies in this review relied on self-reported measures of drinking, with the exception of Peltzer et al. [33], which included BAC. Thus, the findings of this systematic review are subject to recall and social desirability bias associated with self-reported alcohol measurement, shown less reliable than alcohol biomarkers in African cohort studies [52].

The authors made judgements of clinical significance based on the information available, which typically included effect size, changes from baseline drinking rates, population, and setting. However, considerations of clinically meaningful interventions should take into account a wider range of issues, including implementation feasibility, provider buy-in, participant preferences, cost-effectiveness, sustainability, and availability of other interventions [25, 26]. These factors are outside of the scope of the current study, and were not included in the information available to review authors.

In this review, the intervention effect reported is specific to HED. As discussed, a review focused on this pattern of drinking is warranted given the high rates of HED in sub-Saharan Africa. However, the interventions included in this review may have had positive effects on other drinking outcomes not reported in this paper, such as overall alcohol quantity and frequency of consumption. Thus, the findings of this study are not generalizable to other patterns of drinking. We report on other drinking patterns (i.e., AUDIT score, abstinence, drinking quantity, drinking frequency) in a companion meta-analysis [21]. In addition, more than half of studies in this review included content dually focused on alcohol use and other behavior change (e.g., HIV risk reduction). We cannot verify the dosage of intervention content specific to HED compared to other patterns of drinking, or specific to other health behaviors addressed in dual interventions, which could contribute to the varying intervention effects on HED outcomes observed across studies.

### Conclusions

This systematic review found some evidence to suggest non-pharmacological alcohol interventions may reduce HED outcomes in sub-Saharan African settings. However, the clinical significance of statistically significant findings varied, and just over half of studies found no effect at all. Promising interventions included both individual and group approaches, those with an intervention dose of two hours or greater, and a variety of psychosocial approaches. Although MI-based BI showed some promise, the majority of studies that employed this approach reported no change in HED outcomes. In addition, this review highlights an overwhelming focus of the literature on interventions aimed at individual-level, rather than structural-level, change. More research is needed to provide pointed policy and practice recommendations on which interventions work to reduce HED in different sub-Saharan African settings and populations. This review specifically highlights the need for intervention research to: 1) develop and test intervention approaches tailored to the reduction of HED; 2) identify feasible and sustainable BI approaches; and 3) test structural approaches that target social and environmental contributors to HED. To strengthen the alcohol-focused intervention literature in sub-Saharan Africa, research should employ more rigorous designs (i.e., randomized controlled trials), and employ more consistent measurement of HED including the use of alcohol biomarkers.

## Supporting information

S1 Appendix Completed 2009 PRISMA checklist. (DOCX)

S2 Appendix Search strategy table. (DOCX)

S3 Appendix Standardized checklist for the review of full text articles. (DOCX)

S4 Appendix (XLSX)

S5 Appendix Criteria for judging risk of bias in the ‘risk of bias’ assessment tool. (DOCX)

S6 Appendix List of excluded studies with reasons for exclusion. (DOCX)

S7 Appendix Figure summarizing the risk of bias assessment by study and outcome. (DOCX)

S8 Appendix Findings of the risk of bias assessment by study and outcome. (DOCX)
