# Supplementary material for: A systematic review of interventions for reducing heavy episodic drinking in sub-Saharan African settings
Source: PLoS One. 2020 Dec 1;15(12):e0242678. doi: 10.1371/journal.pone.0242678 (PMC7707537; doi:10.1371/journal.pone.0242678)
Supplement: S2 Appendix — (DOCX) [file pone.0242678.s002.docx]

# **S2 APPENDIX:** Search Strategy Table

| **OVID MEDLINE** | **OVID PsycINFO** | **PUBMED** | **EMBASE** | **CINAHL** | **CCTR [Central]** |
| --- | --- | --- | --- | --- | --- |
| **INTERVENTION/ EXPOSURE** | | | | | |
| exp "Alcoholism/prevention and control"  exp “Alcoholism/rehabilitation"  exp “Alcoholism/therapy”  exp "Binge Drinking/prevention and control"  exp Binge Drinking/rehabilitation"  exp Binge Drinking/therapy”  exp "Alcoholic Intoxication/prevention and control"  exp "Alcoholic Intoxication/therapy"  exp "Alcoholic Intoxication/rehabilitation"  exp "Alcohol Drinking/prevention and control"  exp "Alcohol Drinking/rehabilitation"/  exp "Alcohol Drinking/therapy"  interven*.ti,ab. or prevention.ti,ab. or reduce.ti,ab. or reduction.ti,ab. or risk-reduction.ti,ab. or therapy.ti,ab. or counsel*.ti,ab. or rehabilitation.ti,ab.  AND  alcohol*.ti,ab. OR binge drinking.ti,ab. OR “alcohol use”.ti,ab. | exp "Alcoholism/  exp "Alcohol Drinking Patterns/  exp "Alcohol Abuse/  Exp “Binge Drinking/  interven*.ti,ab. or prevention.ti,ab. or reduce.ti,ab. or reduction.ti,ab. or risk-reduction.ti,ab. or therapy.ti,ab. or counsel*.ti,ab. or rehabilitation.ti,ab.  AND  alcohol*.ti,ab. OR binge drinking.ti,ab. OR “alcohol use”.ti,ab. | "Alcoholism/prevention and control"[Mesh]  “Alcoholism/rehabilitation"[Mesh]  “Alcoholism/therapy” [Mesh]  "Binge Drinking/prevention and control"[Mesh]  Binge Drinking/rehabilitation" [Mesh]  Binge Drinking/therapy” [Mesh]  "Alcoholic Intoxication/prevention and control"[Mesh]  "Alcoholic Intoxication/therapy"[Mesh]  "Alcoholic Intoxication/rehabilitation"[Mesh]  "Alcohol Drinking/prevention and control"[Mesh]  "Alcohol Drinking/rehabilitation"[Mesh]  "Alcohol Drinking/therapy"[Mesh]  interven* [tiab] OR prevention [tiab] OR reduce [tiab] OR reduction [tiab] OR “risk-reduction” [tiab] OR therapy [tiab] OR counsel* [tiab] OR rehabilitation [tiab]  AND  alcohol [tiab] OR binge drinking [tiab] OR alcohol use [tiab] | exp "Alcoholism/prevention and control"  exp “Alcoholism/rehabilitation"  exp “Alcoholism/therapy”  exp "Binge Drinking/prevention and control"  exp Binge Drinking/rehabilitation"  exp Binge Drinking/therapy”  exp "Alcoholic Intoxication/prevention and control"  exp "Alcoholic Intoxication/therapy"  exp "Alcoholic Intoxication/rehabilitation"  Drinking behavior/prevention  Drinking behavior/therapy  Alcohol intoxication/prevention  Alcohol intoxication/rehabilitation  Alcohol intoxication/therapy  exp Alcohol consumption/  interven*.ti,ab. or prevention.ti,ab. or reduce.ti,ab. or reduction.ti,ab. or risk-reduction.ti,ab. or therapy.ti,ab. or counsel*.ti,ab. or rehabilitation.ti,ab.  AND  alcohol*.ti,ab. OR binge drinking.ti,ab. OR “alcohol use”.ti,ab. | (MH "Alcoholism/ED/PC/TH/RH")  (MH "Alcohol Rehabilitation Programs")  (MH "Binge Drinking/ED/PC/RH/TH")  (MH "Alcoholic Intoxication/ED/PC/TH/RH")  “interven*” OR “prevention” OR “reduce” OR “reduction” OR “risk-reduction” OR “therapy” OR “counsel*” OR “rehabilitation”  AND  “alcohol*” OR “binge drinking” OR “alcohol use” | MeSH descriptor: [Alcoholism] explode all trees and with qualifier(s): [prevention & control - PC, rehabilitation - RH, therapy - TH]  MeSH descriptor: [Binge Drinking] explode all trees and with qualifier(s): [prevention & control - PC, rehabilitation - RH, therapy - TH]  MeSH descriptor: [Alcoholic Intoxication] explode all trees and with qualifier(s): [prevention & control - PC, rehabilitation - RH, therapy - TH]  MeSH descriptor: [Alcohol Drinking] explode all trees and with qualifier(s): [prevention & control - PC, therapy - TH]  "interven*" or "prevention" or "reduce" or "reduction" or "risk-reduction" or "therapy" or "counsel*" or "rehabilitation" in Trials  "alcohol*" or "binge drinking" or "alcohol use" in Trials |
| **PATIENT/POPULATION** | | | | | |
| exp "Africa South of the Sahara"/  "sub-saharan africa".ti,ab  Cameroon.ti,ab.  Central African Republic .ti,ab.  Chad .ti,ab.  Congo .ti,ab.  Democratic Republic of the Congo .ti,ab.  Equatorial Guinea .ti,ab.  Gabon .ti,ab.  Burundi .ti,ab.  Djibouti .ti,ab.  Eritrea .ti,ab.  Ethiopia .ti,ab.  Kenya .ti,ab.  Rwanda .ti,ab.  Somalia .ti,ab.  South Sudan .ti,ab.  Sudan .ti,ab.  Tanzania .ti,ab.  Uganda .ti,ab.  Angola .ti,ab.  Botswana .ti,ab.  Lesotho .ti,ab.  Malawi .ti,ab.  Mozambique.ti,ab.  Namibia .ti,ab.  South Africa .ti,ab.  Swaziland .ti,ab.  Zambia Zimbabwe .ti,ab.  Benin .ti,ab.  Burkina Faso .ti,ab.  Cape Verde .ti,ab.  Cote d'Ivoire .ti,ab.  Gambia .ti,ab.  Ghana .ti,ab.  Guinea .ti,ab.  Guinea-Bissau .ti,ab.  Liberia .ti,ab.  Mali .ti,ab.  Mauritania .ti,ab.  Niger .ti,ab.  Nigeria .ti,ab.  Senegal .ti,ab.  Sierra Leone .ti,ab.  Togo.ti,ab. | "sub-saharan africa".ti,ab  Cameroon.ti,ab.  Central African Republic .ti,ab.  Chad .ti,ab.  Congo .ti,ab.  Democratic Republic of the Congo .ti,ab.  Equatorial Guinea .ti,ab.  Gabon .ti,ab.  Burundi .ti,ab.  Djibouti .ti,ab.  Eritrea .ti,ab.  Ethiopia .ti,ab.  Kenya .ti,ab.  Rwanda .ti,ab.  Somalia .ti,ab.  South Sudan .ti,ab.  Sudan .ti,ab.  Tanzania .ti,ab.  Uganda .ti,ab.  Angola .ti,ab.  Botswana .ti,ab.  Lesotho .ti,ab.  Malawi .ti,ab.  Mozambique.ti,ab.  Namibia .ti,ab.  South Africa .ti,ab.  Swaziland .ti,ab.  Zambia Zimbabwe .ti,ab.  Benin .ti,ab.  Burkina Faso .ti,ab.  Cape Verde .ti,ab.  Cote d'Ivoire .ti,ab.  Gambia .ti,ab.  Ghana .ti,ab.  Guinea .ti,ab.  Guinea-Bissau .ti,ab.  Liberia .ti,ab.  Mali .ti,ab.  Mauritania .ti,ab.  Niger .ti,ab.  Nigeria .ti,ab.  Senegal .ti,ab.  Sierra Leone .ti,ab.  Togo.ti,ab. | "Africa South of the Sahara"[Mesh Terms]  "sub-saharan africa" [tiab]  Cameroon [tiab]  Central African Republic [tiab]  Chad [tiab]  Congo [tiab]  Democratic Republic of the Congo [tiab]  Equatorial Guinea [tiab]  Gabon [tiab]  Burundi [tiab]  Djibouti [tiab]  Eritrea [tiab]  Ethiopia [tiab]  Kenya [tiab]  Rwanda [tiab]  Somalia [tiab]  South Sudan [tiab]  Sudan [tiab]  Tanzania [tiab]  Uganda [tiab]  Angola [tiab]  Botswana [tiab]  Lesotho [tiab]  Malawi [tiab]  Mozambique [tiab]  Namibia [tiab]  South Africa [tiab]  Swaziland [tiab]  Zambia Zimbabwe [tiab]  Benin [tiab]  Burkina Faso [tiab]  Cape Verde [tiab]  Cote d'Ivoire [tiab]  Gambia [tiab]  Ghana [tiab]  Guinea [tiab]  Guinea-Bissau [tiab]  Liberia [tiab]  Mali [tiab]  Mauritania [tiab]  Niger [tiab]  Nigeria [tiab]  Senegal [tiab]  Sierra Leone [tiab]  Togo [tiab] | exp "Africa South of the Sahara"/  "sub-saharan africa".ti,ab  Cameroon.ti,ab.  Central African Republic .ti,ab.  Chad .ti,ab.  Congo .ti,ab.  Democratic Republic of the Congo .ti,ab.  Equatorial Guinea .ti,ab.  Gabon .ti,ab.  Burundi .ti,ab.  Djibouti .ti,ab.  Eritrea .ti,ab.  Ethiopia .ti,ab.  Kenya .ti,ab.  Rwanda .ti,ab.  Somalia .ti,ab.  South Sudan .ti,ab.  Sudan .ti,ab.  Tanzania .ti,ab.  Uganda .ti,ab.  Angola .ti,ab.  Botswana .ti,ab.  Lesotho .ti,ab.  Malawi .ti,ab.  Mozambique.ti,ab.  Namibia .ti,ab.  South Africa .ti,ab.  Swaziland .ti,ab.  Zambia Zimbabwe .ti,ab.  Benin .ti,ab.  Burkina Faso .ti,ab.  Cape Verde .ti,ab.  Cote d'Ivoire .ti,ab.  Gambia .ti,ab.  Ghana .ti,ab.  Guinea .ti,ab.  Guinea-Bissau .ti,ab.  Liberia .ti,ab.  Mali .ti,ab.  Mauritania .ti,ab.  Niger .ti,ab.  Nigeria .ti,ab.  Senegal .ti,ab.  Sierra Leone .ti,ab.  Togo.ti,ab. | (MH "Africa South of the Sahara")  "sub-saharan africa"  “Subsaharan Africa”  “Cameroon”  “Central African Republic”  “Chad”  “Congo”  “Democratic Republic of the Congo”  “Equatorial Guinea”  “Gabon”  “Burundi”  “Djibouti”  “Eritrea”  “Ethiopia”  “Kenya”  “Rwanda”  “Somalia”  “South Sudan”  “Sudan”  “Tanzania”  “Uganda”  “Angola”  “Botswana”  “Lesotho”  “Malawi”  “Mozambique”  “Namibia”  “South Africa”  “Swaziland”  “Zambia Zimbabwe”  “Benin”  “Burkina Faso”  “Cape Verde”  “Cote d'Ivoire”  “Gambia”  “Ghana”  “Guinea”  “Guinea-Bissau”  “Liberia”  “Mali”  “Mauritania”  “Niger”  “Nigeria”  “Senegal”  “Sierra Leone”  “Togo” | MeSH descriptor: [Africa South of the Sahara] explode all trees  "Subsaharan Africa" in Trials  "sub-saharan africa" or "Cameroon" or "Central African Republic" or "Chad" or "Congo" or "Democratic Republic of the Congo" or "Equatorial Guinea" or "Gabon" or "Burundi" or "Djibouti" or "Eritrea" or "Ethiopia" or "Kenya" or "Rwanda" or "Somalia" or "South Sudan" or "Sudan" or "Tanzania" or "Uganda" or "Angola" or "Botswana" or "Lesotho" or "Malawi" or "Mozambique" or "Namibia" or "South Africa" or "Swaziland" or "Zambia Zimbabwe" or "Benin" or "Burkina Faso" or "Cape Verde" or "Cote d'Ivoire" or "Gambia" or "Ghana" or "Guinea" or "Guinea-Bissau" or "Liberia" or "Mali" or "Mauritania" or "Niger" or "Nigeria" or "Senegal" or "Sierra Leone" or "Togo":ti,ab,kw (Word variations have been searched) in Trials |
| **LIMITS** | | | | | |
| Limit to publication types:  "Randomized Controlled Trial" OR Clinical Trial  exp Non-Randomized Controlled Trials as Topic/  randomized.ti,ab. OR quasi-experimental.ti,ab. OR quasi experimental.ti,ab. OR random allocation.ti,ab. OR comparative study.ti,ab.  Humans  English | Limit to publication types:  "0200 clinical case study" or "0300 clinical trial" or "0400 empirical study" or "0410 experimental replication" or "0430 followup study" or "0450 longitudinal study" or "0451 prospective study" or "0453 retrospective study" or "0600 field study" or "0700 interview" or "0750 focus group" or 1400 nonclinical case study or 1600 qualitative study or 1800 quantitative study or 2100 treatment outcome  randomized.ti,ab. OR quasi-experimental.ti,ab. OR quasi experimental.ti,ab. OR random allocation.ti,ab. OR comparative study.ti,ab.  Humans  English | "Randomized Controlled Trial"[Publication Type] OR "Non-Randomized Controlled Trials as Topic"[Mesh] OR "randomized" [tiab] OR "quasi-experimental" [tiab] OR "quasi experimental" [tiab] OR “random allocation” [tiab] OR “comparative study” [tiab] OR Clinical Trial[ptyp]  Humans  English | Limit to publication types:  clinical trial or randomized controlled trial or controlled clinical trial or multicenter study or phase 1 clinical trial or phase 2 clinical trial or phase 3 clinical trial or phase 4 clinical trial  exp Non-Randomized Controlled Trials as Topic/  randomized.ti,ab. OR quasi-experimental.ti,ab. OR quasi experimental.ti,ab. OR random allocation.ti,ab. OR comparative study.ti,ab.  Humans  English | (MH "Intervention Trials") OR (MH "Experimental Studies") OR (MH "Randomized Controlled Trials") OR (MH "Quasi-Experimental Studies") OR (MH "Clinical Trials") OR (MH "Pretest-Posttest Control Group Design") OR (MH "Comparative Studies") OR "Non-Randomized Controlled Trials as Topic"  (TI "random*”) OR  (AB "random*”) OR  (TI "quasi-experimental") OR  (AB "quasi-experimental") OR  (TI "quasi experimental") OR  (AB "quasi experimental") OR  (TI “random allocation”) OR  (AB “random allocation”) OR  (TI “comparative study”) OR  (AB “comparative study”) OR  (TI “experiment*”) OR  (AB “experiment*”)  Humans  English |  |
