# Supplementary material for: A systematic review of interventions for reducing heavy episodic drinking in sub-Saharan African settings
Source: PLoS One. 2020 Dec 1;15(12):e0242678. doi: 10.1371/journal.pone.0242678 (PMC7707537; doi:10.1371/journal.pone.0242678)
Supplement: S3 Appendix — (DOCX) [file pone.0242678.s003.docx]

# **S3 APPENDIX.** Standardized Checklist for the Review of Full Text Articles

| **Inclusion Criteria** | **Description** |
| --- | --- |
| SSA | Study took place in sub-Saharan Africa |
| Intervention study | Study is testing an intervention to reduce alcohol use **Exclusion:** Intervention is a pharmacological alcohol intervention; alcohol reduction is not a primary goal of the intervention; focus of alcohol reduction is only in the context of sex |
| Controlled trial | Study design includes intervention and comparison group, including randomized controlled trials (RCTs), cluster randomized controlled groups (CRCT), and quasi-experimental controlled groups (non-randomized eligible) **Exclusion:** pre/post-test single armed studies with no comparison group; non-intervention designs such as cross-sectional and observational studies |
| Binge/Heavy Episodic Drinking Outcome | Binge drinking: e.g., four drinks within two hours for women or five drinks within two hours for men Heavy episodic drinking: e.g., at least 60 grams of pure alcohol on at least one occasion in the past 30 days Measures indicative of HED or binge drinking: (e.g., high BAC, frequency of intoxication in past 30 days)  **Exclusion:** Drinking quantity; drinking frequency; AUDIT score; AUDIT categories: Harmful/hazardous drinking; Dependence; ASSIST scores; CAGE scores; alcohol abstinence (any drinking vs. no drinking) |
| Comparator | Intervention unrelated to alcohol, usual care for alcohol or other services, brief feedback on an alcohol screening tool, alcohol or other informational materials, wait-list, and nothing **Exclusion:** no comparator condition; comparator was another evidence-based or ‘bona-fide’ alcohol intervention (i.e., non-inferiority trial) |
