# Supplementary material for: A systematic review of interventions for reducing heavy episodic drinking in sub-Saharan African settings
Source: PLoS One. 2020 Dec 1;15(12):e0242678. doi: 10.1371/journal.pone.0242678 (PMC7707537; doi:10.1371/journal.pone.0242678)
Supplement: S6 Appendix — (DOCX) [file pone.0242678.s006.docx]

# **S6 APPENDIX:** List of Excluded Studies with Reasons for Exclusion

| **Study Reference** | | **Reason For Exclusion** |
| --- | --- | --- |
|  | Ayodapo AO, Olukokun TAV. Lifestyle counselling and behavioural change: role among adult hypertensives in a rural tertiary institution. *S Afr Fam Pract* 2019; 61(3): 91-6. | Not a specified heavy episodic drinking outcome |
|  | Bachanas P, Kidder D, Medley A, et al. Delivering prevention interventions to people living with HIV in clinical care settings: results of a cluster randomized trial in Kenya, Namibia, and Tanzania*. AIDS Behav* 2016; 20(9): 2110-8. doi: 10.1007/s10461-016-1349-2. | Not a specified heavy episodic drinking outcome |
|  | Baker L. Determining the efficacy of choices: A group-format multicomponent alcohol intervention. *Dissertation Abstracts International: Section B: The Sciences and Engineering* 2015; 76. | Not in sub Saharan Africa |
|  | Ben-Arie O, Swartz L, George G. The compulsory treatment of alcoholic drunken drivers referred by the courts: A 7 to 9 years outcome study. *Int J Law Psychiatry* 1986; 8(2): 229-35. | Not an alcohol intervention study |
|  | Bodewes C. Chang'AA drinking in Kibera slum: The harmful effect of contemporary changes in the production and consumption of traditional spirits. *Afr J Drug Alcohol Stud* 2010; 9(1): 49-56. | Not an alcohol intervention study |
|  | Brown JC, Gardner-Lubbe S, Lambert MI, Van Mechelen W, Verhagen E. The BokSmart intervention programme is associated with improvements in injury prevention behaviours of rugby union players: An ecological cross-sectional study. *Inj Prev* 2015; 21(3): 173-8. | Not an alcohol intervention study |
|  | Carney T, Johnson K, Carrico A, Myers B. Acceptability and feasibility of a brief substance use intervention for adolescents in Cape Town, South Africa: a pilot study. *Int J Psychol*. 2020;13. | Not a specified heavy episodic drinking outcome; single armed trial |
|  | Carney T, Petersen WPM, Parry CD. Ithubalethu-Intervention to Address Drug Use and Sexual HIV Risk Patterns among Female Commercial Sex Workers in Durban, South Africa. *J Psychoactive Drugs*, 2016 48(4):303-9 | Intervention trial, but a single armed study |
|  | Chaudhury S, Brown FL, Kirk CM, et al. Exploring the potential of a family-based prevention intervention to reduce alcohol use and violence within HIV-affected families in Rwanda. *AIDS care*, 2016; 28 Suppl 2:118-29 | Not a specified heavy episodic drinking outcome |
|  | Clair VM, Musau A, Frank E, Ndetei D. (2017). The computer-based drug and alcohol training assessment in Kenya. *Drug and Alcohol*  *Dependence*, 2017; 171, e2–e226. doi:10.1016/j.drugalcdep.2016.08.129 | Control group received an alcohol intervention (determined by discussion with the study author) |
|  | Clair, V. M., . V., Musau, A., Frank, E., & Ndetei, D. (2016). Online learning improves substance use care in Kenya: randomized control trial results and implications*. Ann Global Health*, 2017 82(3), 319–327. | Control group received an alcohol intervention (determined by discussion with the study author) |
|  | Deveau CS. HIV prevention among drug and alcohol users: Models of intervention in Kenya. *Afr J Drug Alcohol Stud* 2008; 7(2): 113-26. | Not an alcohol intervention study |
|  | Duwell MM, Knowlton AR, Nachega JB, et al. Patient-nominated, community-based HIV treatment supporters: patient perspectives, feasibility, challenges, and factors for success in HIV-infected South African adults. *AIDS Patient Care STDS*, 2013; 27(2):96-102. doi: 10.1089/apc.2012.0348. | Not an alcohol intervention study |
|  | Emenyonu NI, Fatch R, Muyindike WR, Kekibiina A, Woolf-King S, Hahn JA. Randomized study of assessment effects on alcohol use by persons with HIV in rural Uganda. *J Stud Alcohol Drugs*, 2017 78(2):296-305. | Not an alcohol intervention study |
|  | Fitzgerald AM, Stanton BF, Terreri N, et al. Use of Western-based HIV risk-reduction interventions targeting adolescents in an African setting. *J Adolesc Health* 1999; 25(1): 52-61. | Non-primary paper of another paper |
|  | Fritz K, McFarland W, Wyrod R, et al. Evaluation of a peer network-based sexual risk reduction intervention for men in beer halls in Zimbabwe: results from a randomized controlled trial. *AIDS Behav* 2011; 15(8): 1732-44. | Not a specified heavy episodic drinking outcome |
|  | Gichane MW, Wechsberg WM, Ndirangu J, et al. Implementation science outcomes of a gender-focused HIV and alcohol risk-reduction intervention in usual-care settings in South Africa. *Drug Alcohol Depend*. 2020;215 (no pagination)(108206). | Non-primary paper of another paper; implementation science outcome paper for Wechsberg, 2019 |
|  | Giusto A, Green EP, Simmons RA, Ayuku D, Patel P, Puffer ES. A multiple baseline study of a brief alcohol reduction and family engagement intervention for fathers in Kenya. *J Consult Clin Psychol*. 2020;88(8):708-25. | Design: Single cohort, cross-over design; not a specified heavy episodic drinking outcome |
|  | Hahn JA, Emenyonu NI, Fatch R, Muyindike WR, Kekibiina A, Woolf-King S. Randomized study of assessment reactivity in persons with HIV in rural Uganda measured using self-report and phosphatidylethanol. Research Society on Alcoholism; 2015 June; San Antonio, TX: Alcoholism; 2015. | Review study |
|  | Hailu FB, Moen A, Hjortdahl P. Diabetes self-management education (DSME) - “ Effect on knowledge, self-care behavior, and self-efficacy among type 2 diabetes patients in Ethiopia: a controlled clinical trial. *Diabetes Metab Syndr Obes*. 2019;12:2489‐99. | Not an alcohol intervention; alcohol only secondary aim |
|  | Harder VS, Musau AM, Musyimi CW, Ndetei DM, Mutiso VN. A randomized clinical trial of mobile phone motivational interviewing for alcohol use problems in Kenya. *Addiction*. 2020;115(6):1050-60. | Not a specified heavy episodic drinking outcome |
|  | Harris SK, Seale D, Pande Y, et al. 25. Testing feasibility/acceptability and initial outcomes of a spiritually-based character strengths training curriculum to enhance resilience and reduce substance use rates among Zambian adolescents. *J Adolesc Health*. 2020;66 (2 Supplement):S13-S4. | Non-primary paper of another paper |
|  | Huis In 't Veld D, Ensoy-Musoro C, Pengpid S, Peltzer K, Colebunders R. The efficacy of a brief intervention to reduce alcohol use in persons with HIV in South Africa, a randomized clinical trial. *PLoS One.* 2019;14(8):e0220799. | Not a specified heavy episodic drinking outcome |
|  | Jemmott JB III, Zhang J, Jemmott LS, et al. Intervention increases physical activity and healthful diet among South African adolescents over 54 months: A randomized controlled trial. *J Adolesc Health*. 2019;65(1):139-46. | Not an alcohol intervention; alcohol only secondary aim |
|  | Kalichman SC, Cain D, Eaton L, Jooste S, Simbayi LC. Randomized clinical trial of brief risk reduction counseling for sexually transmitted infection clinic patients in Cape Town, South Africa. *Am J Public Health* 2011; 101(9): e9-e17. | Not a specified heavy episodic drinking outcome |
|  | Kalichman SC, Mathews C, Banas E, Kalichman MO. Stigma management intervention to improve antiretroviral therapy adherence: Phase-I test of concept trial, Cape Town South Africa. *Glob Public Health*. 2019;14(8):1059-74. | Not a specified heavy episodic drinking outcome |
|  | Kalichman SC, Simbayi LC, Cain D, et al. Randomized community-level HIV prevention intervention trial for men who drink in South African alcohol-serving venues. *Eur J Public Health* 2014; 24(5): 833-9. | Not a specified heavy episodic drinking outcome |
|  | Kalichman SC, Simbayi LC, Cloete A, et al. Integrated gender-based violence and HIV Risk reduction intervention for South African men: results of a quasi-experimental field trial. *Prev Sci* 2009; 10(3): 260-9. | Not a specified heavy episodic drinking outcome |
|  | Kalichman SC, Simbayi LC, Vermaak R, Cain D, Jooste S, Peltzer K. HIV/AIDS risk reduction counseling for alcohol using sexually transmitted infections clinic patients in Cape Town, South Africa. *J Acquir Immune Defic Syndr* 2007; 44(5): 594-600. | Not a specified heavy episodic drinking outcome |
|  | Kalichman SC, Simbayi LC, Vermaak R, et al. Randomized trial of a community-based alcohol-related HIV risk-reduction intervention for men and women in Cape Town South Africa. *Ann Behav Med* 2008; 36(3): 270-9. | Not a specified heavy episodic drinking outcome |
|  | Karnell AP, Cupp PK, Zimmerman RS, Feist-Price S, Bennie T. Efficacy of an American alcohol and HIV prevention curriculum adapted for use in South Africa: Results of a pilot study in five township schools. *AIDS Educ Prev* 2006; 18(4): 295-310. | Not a specified heavy episodic drinking outcome |
|  | Lasebikan V, Ola BA, Ayinde OO. Effectiveness of alcohol, smoking, and substance involvement screening test-linked brief intervention on harmful and hazardous alcohol use in Nigerian semirural communities: A non-randomized intervention study. *Front Psychiatry* 2017; 8(50): 1-7. | Intervention trial, but a single armed study |
|  | Lasebikan VO, Lasebikan GL. Translation of the 12-step alcoholic anonymous from English language to Yoruba language and its application within the Yoruba culture among patients with alcohol use disorder in Nigeria. *Ment Health Relig Cult* 2017; 20(9): 898-910. | Not a specified heavy episodic drinking outcome |
|  | Lau WK, Leung PP, Chung CL. Effects of the satir model on mental health: a randomized controlled trial. *Res Soc Work Pract*. 2019;29(7):775‐85. | Not in Sub-Saharan Africa |
|  | Louwagie G, Morojele N, Siddiqi K, et al. Improving TB outcomes by modifying life-style behaviours through brief motivational interviewing and text-messaging-a feasibility trial. *Trop Med Int Health* 2017; 22 (Supplement 1): 267. | Not a specified heavy episodic drinking outcome |
|  | Louwagie GM, Morojele N, Siddiqi K, et al. Addressing tobacco smoking and drinking to improve TB treatment outcomes, in South Africa: a feasibility study of the ProLife program [published online ahead of print, 2019 Jun 24]. *Transl Behav Med*. 2019;ibz100. doi:10.1093/tbm/ibz100 | Design: Not a RCT, alcohol intervention but reporting on feasibility outcomes |
|  | Madhombiro M, Dube B, Dube M, et al. Intervention for alcohol use disorders at an HIV care clinic in Harare: a pilot and feasibility study. *Addict Sci Clin Pract*. 2019;14(1):16. Published 2019 Apr 5. doi:10.1186/s13722-019-0143-7 | Not a specified heavy episodic drinking outcome; comparator also not eligible (another alcohol intervention) |
|  | Manyaapelo T, Borne BVD, Ruiter RAC, Sifunda S, Reddy P. Effectiveness of a health behavioural intervention aimed at reduction of risky sexual behaviours among young men in the Kwazulu-Natal province, South Africa. *Int J Environ Res Public Health*. 2019;16 (11) (no pagination)(1938). | Not a specified heavy episodic drinking outcome |
|  | Marais S, Jordaan E, Viljoen D, Olivier L, Waal JD, Poole C. The effect of brief interventions on the drinking behaviour of pregnant women in a high-risk rural South African community: A cluster randomised trial. *Early Child Dev Care* 2011; 181(4): 463-74. | Not a specified heavy episodic drinking outcome |
|  | Moriarty AS, Louwagie GM, Mdege ND, et al. ImPROving TB outcomes by modifying LIFE-style behaviours through a brief motivational intervention followed by short text messages (ProLife): Study protocol for a randomised controlled trial. *Trials*. 2019;20(1). | Study Protocol |
|  | Moscoe E, Agot K, Thirumurthy H. Effect of a prize-linked Savings intervention on savings and healthy behaviors among men in Kenya: a randomized clinical trial. *JAMA Netw Open*. 2019;2(9):e1911162. | Not a specified heavy episodic drinking outcome; measured expenditure on alcohol |
|  | Motamedi M, Caldwell L, Wegner L, Smith E, Jones D. Girls just want to know where to have fun: Preventing substance use initiation in an under-resourced community in South Africa through HealthWise. *Prev Sci* 2016; 17(6): 700-9. | Not a specified heavy episodic drinking outcome |
|  | Munodawafa D, Marty PJ, Gwede C. Effectiveness of health instruction provided by student nurses in rural secondary schools of Zimbabwe: a feasibility study. *Int J Nurs Stud* 1995; 32(1): 27-38. | Not a specified heavy episodic drinking outcome |
|  | Muriungi SK, Ndetei DM. Effectiveness of psycho-education on depression, hopelessness, suicidality, anxiety and substance use among basic diploma students at Kenya Medical Training College. *S Afr J Psych* 2013; 19(2): 41-50. | Not a specified heavy episodic drinking outcome |
|  | Murray LK, Kane JC, Glass N, et al. Effectiveness of the common elements treatment approach (CETA) in reducing intimate partner violence and hazardous alcohol use in Zambia (VATU): a randomized controlled trial. *PLoS Med*. 2020;17(4):e1003056. | Not a specified heavy episodic drinking outcome |
|  | Mutiso V, Pike K, Musyimi C, et al. Feasibility and effectiveness of nurses and clinical officers in implementing the WHO mhGAP intervention guide: Pilot study in Makueni County, Kenya. *Gen Hosp Psychiatry*. 2019;59:20-9. | Design: uncontrolled, prospective, continuous recruitment design, intervention study (no comparison group); testing the WHO mental health Global Action Programme Intervention Guide -- not a specified heavy episodic drinking outcome |
|  | Myers B, Carney T, Browne FA, Wechsberg WM. A trauma-informed substance use and sexual risk reduction intervention for young South African women: a mixed-methods feasibility study. *BMJ Open*. 2019;9(2). | Design: Feasibility trial of an alcohol intervention but single armed mixed methods evaluation focused on implementation and feasibility |
|  | Myers B, Stein DJ, Sorsdahl K. Readiness to change is a predictor of reduced substance use involvement: findings from a randomized controlled trial of patients attending South African emergency departments. *Drug and alcohol dependence Conference: 2016 annual meeting of the college on problems of drug dependence, CPDD 2016 United states* 2017; 171: e150. | Not a specified heavy episodic drinking outcome |
|  | Nadkarni A, Weiss HA, Velleman R, et al. Feasibility, acceptability and cost‐effectiveness of a brief, lay counsellor‐delivered psychological treatment for men with alcohol dependence in primary care: an exploratory randomized controlled trial. *Addiction*. 2019;114(7):1192‐203. | Not in Sub-Saharan Africa |
|  | Papas RK, Gakinya BN, Mwaniki MM, Lee H, Keter AK, Klein DA, et al. Successful treatment outcomes from a stage 2 randomized clinical trial of CBT to reduce alcohol use among HIV-infected outpatients in Western Kenya. Alcoholism: Clinical and Experimental Research. 2017;41:253A. | Non-primary paper of another paper |
|  | Papas RK, Sidle JE, Gakinya BN, Baliddawa JB, Martino S, Mwaniki MM. Treatment outcomes of a Stage 1 cognitive-behavioral trial to reduce alcohol use among HIV-infected outpatients in western Kenya. Addiction 2011; 106(12) :2156-66. doi: 10.1111/j.1360-0443.2011.03518.x. | Not a specified heavy episodic drinking outcome |
|  | Papas RK, Sidle JE, Martino S, et al. Systematic cultural adaptation of cognitive-behavioral therapy to reduce alcohol use among HIV-infected outpatients in western Kenya. *AIDS Behav* 2010; 14(3): 669-78. | Intervention trial, but single-armed design |
|  | Parcesepe AM, L. Engle KL, Martin SL, et al. The impact of an alcohol harm reduction intervention on interpersonal violence and engagement in sex work among female sex workers in Mombasa, Kenya: Results from a randomized controlled trial. *Drug Alcohol Depend* 2016; 161: 21-8. | Not a specified heavy episodic drinking outcome |
|  | Parry CDH, Carney T, Petersen-Williams P. Reducing substance use and risky sexual behaviour among drug users in Durban, South Africa: Assessing the impact of community-level risk-reduction interventions. *SAHARA J* 2017; 14(1): 110-7. | Intervention trial, but single-armed design |
|  | Peltzer K. Screening and brief advice for risky substance users among charismatic and main stream churches in South Africa. *J Psychol Afr* 2008; 18(4): 631-6. | Not a specified heavy episodic drinking outcome |
|  | Peltzer K, Babayigit S, Rodriguez VJ, Jean J, Sifunda S, Jones DL. Effect of a multicomponent behavioural PMTCT cluster randomised controlled trial on HIV stigma reduction among perinatal HIV positive women in Mpumalanga province, South Africa. *SAHARA J* 2018; 15(1): 80-8. | Not a specified heavy episodic drinking outcome |
|  | Peltzer K, Tabane C, Matseke G, Simbayi L. Lay counsellor-based risk reduction intervention with HIV negative diagnosed patients at HIV counselling and testing sites in a rural South African setting. *J Psychol Afr* 2009; 19(4): 541-8. | Intervention trial, but single-armed design |
|  | Peltzer K, Tabane C, Matseke G, Simbayi L. Lay counsellor-based risk reduction intervention with HIV positive diagnosed patients at public HIV counselling and testing sites in Mpumalanga, South Africa. *Eval Program Plann* 2010; 33(4): 379-85. | Intervention trial, but single-armed design |
|  | Perry CL. Prevention of alcohol use and abuse in adolescence: Teacher- vs peer-led intervention. *Crisis* 1989; 10(1): 52-61. | Intervention trial, but single-armed design |
|  | Perry CL, Grant M, Ernberg G, et al. WHO Collaborative Study on Alcohol Education and Young People: outcomes of a four-country pilot study. *Int J Addict* 1989; 24(12): 1145-71. | Intervention trial, but single-armed design |
|  | Petersen I, Bhana A, Fairall LR, et al. Evaluation of a collaborative care model for integrated primary care of common mental disorders comorbid with chronic conditions in South Africa. *BMC Psychiatry*. 2019;19(1)107. | Not an alcohol reduction intervention; focused on task-shifting and identification of Alcohol Use Disorders/Depression |
|  | Pitpitan EV, Kalichman SC, Garcia RL, Cain D, Eaton LA, Simbayi LC. Mediators of behavior change resulting from a sexual risk reduction intervention for STI patients, Cape Town, South Africa. *J Behav Med* 2015; 38(2): 194-203. | Not a specified heavy episodic drinking outcome |
|  | Ramarumo M, Peltzer K, Khoza LB. Screening and brief intervention of alcohol problems in primary care in South African: A brief report. *J Psychol Afr* 2016; 26(1): 78-80. | Not a specified heavy episodic drinking outcome |
|  | Rotheram-Borus MJ, Roux IML, Tomlinson M, et al. Philani Plus (+): a Mentor Mother community health worker home visiting program to improve maternal and infants' outcomes. *Prev Sci* 2011; 12(4): 372-88. | Not a specified heavy episodic drinking outcome |
|  | Rotheram-Borus MJ, Tomlinson M, Durkin A, Baird K, DeCelles J, Swendeman D. Feasibility of using soccer and job training to prevent drug abuse and HIV. AIDS Behav. 2016;20(9):1841–50. doi:10.1007/s10461-015-1262-0 | Not a specified heavy episodic drinking outcome |
|  | Rotheram-Borus MJ, Tomlinson M, Roux IL, Stein JA. Alcohol use, partner violence, and depression: a cluster randomized controlled trial among urban South African mothers over 3 years. Am J Prev Med, 2015; 49(5):715-25. | Not a specified heavy episodic drinking outcome |
|  | Santos, G. M. S. Substance use and alcohol among key populations at risk for HIV: Novel approaches in intervention development and evaluation [Dissertation Empirical Study; Quantitative Study]; 2015. | Not an alcohol intervention study |
|  | Seale JP, Hadfield K, Seale DM, Pande Y, Lewis TM, Harris SK. The effects of a spiritually-based character strengths training curriculum on alcohol use among Zambian youth: a randomized controlled trial. *Alcohol Clin Exp Res*. 2020;44 (Supplement 1):144A. | Not an alcohol intervention; alcohol only secondary aim |
|  | Senyonyi RM, Underwood LA, Suarez E, Musisi S, Grande TL. Cognitive behavioral therapy group intervention for HIV transmission risk behavior in perinatally infected adolescents. *Health* 2012; 4(12): 1334-45. | Not a specified heavy episodic drinking outcome |
|  | Sheikh W, Paul R, Banda H, Agath K, Luty J. Impact of brief relapse prevention intervention in patients with alcohol dependence in Zambia. *J Subst Use* 2017; 22(1): 113-7. | Not a specified heavy episodic drinking outcome |
|  | Smith EA, Palen LA, Caldwell LL, et al. Substance use and sexual risk prevention in Cape Town, South Africa: an evaluation of the HealthWise program. Prev Sci 2008; 9(4): 311-21. doi: 10.1007/s11121-008-0103-z. | Not a specified heavy episodic drinking outcome |
|  | Sorsdahl K, Myers B, Ward CL, et al. Adapting a blended motivational interviewing and problem-solving intervention to address risky substance use amongst South Africans. *Psychother Res* 2015; 25(4): 435-44. | Intervention trial, but single-armed design |
|  | Sorsdahl K, Stein D, Corrigall J, et al. The efficacy of a blended motivational interviewing and problem solving therapy intervention to reduce substance use among patients presenting for emergency services in South Africa: A randomized controlled trial. *Subst Abuse Treat Prev Policy* 2015; 10. | Not a specified heavy episodic drinking outcome |
|  | Sorsdahl K, Williams PP, Everett-Murphy K, et al. Feasibility and preliminary responses to a screening and brief intervention program for maternal mental disorders within the context of primary care. *Community Ment Health J* 2015; 51(8): 962-9. | Not a specified heavy episodic drinking outcome |
|  | Stanton BF, Li X, Kahihuata J, et al. Increased protected sex and abstinence among Namibian youth following a HIV risk-reduction intervention: a randomized, longitudinal study. AIDS 1998; 12(18): 2473-80. | Not a specified heavy episodic drinking outcome |
|  | Staton C, Vissoci JRN, Swahn M, Hirshon JM, Bartlett J, Mmbaga B. Feasibility of a pragmatic randomized adaptive clinical trial of a brief intervention for alcohol use Inmoshi, Tanzania. Conference abstract from the 42nd Annual RSA Scientific Meeting June 22-26, 2019. *Alcohol Clin Exp Res*. 2019;43 (Supplement 1):80A. | Not a specified heavy episodic drinking outcome |
|  | Takahashi R, Wilunda C, Magutah K, Mwaura-Tenambergen W, Atwoli L, Perngparn U. Evaluation of alcohol screening and community-based brief interventions in rural Western Kenya: a quasi-experimental study. Alcohol Alcohol 2018; 53(1):121-128. doi: 10.1093/alcalc/agx083. | Not a specified heavy episodic drinking outcome |
|  | Tang A, Hamunime N, Adams R, et al. Introduction of an alcohol-related electronic screening and brief intervention (eSBI) program to reduce hazardous alcohol consumption in Namibia's antiretroviral treatment (ART) program. *AIDS and Behav*. 2019;23(11):3078-92. | Design: Single armed mixed methods evaluation focused on implementation and feasibility |
|  | Timol F, Vawda M, Bhana A, Moolman B, Makoae M, Swartz S. Addressing adolescents' risk and protective factors related to risky behaviours: Findings from a school-based peer-education evaluation in the Western Cape. *SAHARA J* 2016; 13(1): 197-207. | Not a specified heavy episodic drinking outcome |
|  | Wandera B, Tumwesigye NM, Nankabirwa JI, et al. Efficacy of a single, brief alcohol reduction intervention among men and women living with HIV/AIDS and using alcohol in Kampala, Uganda: a randomized trial. J Int Assoc Provid AIDS Care, 2017; 16(3):276-285. doi: 10.1177/2325957416649669 | Not a specified heavy episodic drinking outcome |
|  | Ward CL, Mertens JR, Bresick GF, Little F, Weisner CM. Screening and brief intervention for substance misuse: does it reduce aggression and HIV-related risk behaviours? *Alcohol Alcohol* 2015; 50(3): 302-9. | Not a specified heavy episodic drinking outcome |
|  | Wechsberg W, Zule W, Van Der Horst C, et al. Combination prevention for women who use alcohol in South Africa: outcomes from the Women's Health CoOp Plus Study in Pretoria, South Africa. *Journal of the International AIDS Society; 22nd International AIDS Conference Abstract Supplement* 2018; 21. | Non-primary paper of another paper |
|  | Wechsberg WM, Luseno WK, Karg RS, et al. Alcohol, cannabis, and methamphetamine use and other risk behaviours among Black and Coloured South African women: a small randomized trial in the Western Cape. *Int J Drug Policy* 2008; 19(2): 130-9. | Intervention trial, but single-armed design |
|  | Wechsberg WM, Luseno WK, Lam WKK, Parry CDH, Morojele NK. Substance use, sexual risk, and violence: HIV prevention intervention with sex workers in pretoria. *AIDS Behav* 2006; 10(2): 131-7. | Intervention trial, but single-armed design |
|  | Wechsberg WM, Zule WS, El-Bassel N, et al. The male factor: outcomes from a cluster randomized field experiment with a couples-based HIV prevention intervention in a South African township. *Drug Alcohol Depend* 2016; 161(1): 307-15. | Control group was an alcohol intervention |
|  | World Health Organization. Programme on Substance Abuse: Project on identificaiton and mangement of alcohol-related problems: World Health Organization, 1992. | Not a specified heavy episodic drinking outcome |
|  | WHO Brief Intervention Study Team. A cross-national trial of brief interventions with heavy drinkers. *Am J Public Health* 1996; 86(7): 948-55. | Non-primary paper of another paper |
|  | Williams PP, Carney T, Pluddemann A, Parry CD. Intervening to identify and reduce drug use and sexual HIV risk patterns among men who have sex with men in three provinces in South Africa. *J Subst Use* 2014; 19(1-2): 141-6. | Not a specified heavy episodic drinking outcome |
|  | Zule W, Myers B, Carney T, Novak SP, McCormick K, Wechsberg WM. Alcohol and drug use outcomes among vulnerable women living with HIV: results from the Western Cape Women's Health CoOp. AIDS Care 2014; 26(12): 1494-9. doi: 10.1080/09540121.2014.933769 | Not a specified heavy episodic drinking outcome |
