# Supplementary figures and images for: A systematic review of interventions for reducing heavy episodic drinking in sub-Saharan African settings
Source: PLoS One. 2020 Dec 1;15(12):e0242678. doi: 10.1371/journal.pone.0242678 (PMC7707537; doi:10.1371/journal.pone.0242678)

# **S7 APPENDIX:** Figure Summarizing the Risk of Bias Assessment by Study and Outcome

**
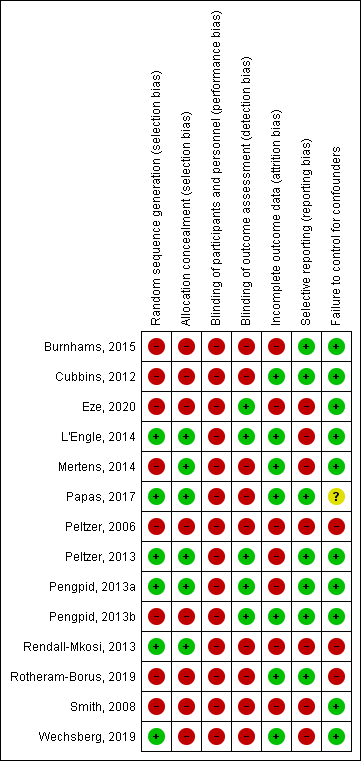

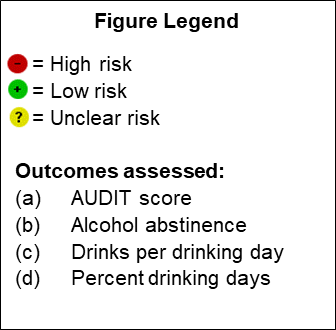
**

Supplement: S7 Appendix — (DOCX) [file pone.0242678.s007.docx]
