# Supplementary material for: A systematic review of interventions for reducing heavy episodic drinking in sub-Saharan African settings
Source: PLoS One. 2020 Dec 1;15(12):e0242678. doi: 10.1371/journal.pone.0242678 (PMC7707537; doi:10.1371/journal.pone.0242678)
Supplement: S8 Appendix — (DOCX) [file pone.0242678.s008.docx]

# **S8 APPENDIX:** Findings of the Risk of Bias Assessment by Study and Outcome

***Burnhams 2015***

| **Bias** | **Authors' judgement** | **Support for judgement** |
| --- | --- | --- |
| Random sequence generation (selection bias) | High risk | Different randomization methods used across clusters, one coin toss, but not enough information on the second arm to judge |
| Allocation concealment (selection bias) | High risk | Different randomization methods used across clusters, including allocation concealment, coin toss for some groups and "done by the principal investigator and biostatistician" |
| Blinding of participants and personnel (performance bias) | High risk | Participants blinded except for length of session; personnel not blinded |
| Blinding of outcome assessment (detection bias) | High risk | Blinding of outcome assessment not reported |
| Incomplete outcome data (attrition bias) | High risk | Loss to follow-up > 20% |
| Selective reporting (reporting bias) | Low risk | Registered with Pan-African Control Trial Registry: 201301000458308 |
| Failure to control for confounders | Low risk | Outcomes adjusted for division, repeated measures, and clustering |

***Cubbins, 2012***

| **Bias** | **Authors' judgement** | **Support for judgement** |
| --- | --- | --- |
| Random sequence generation (selection bias) | High risk | Sample communities were paired based on geographic proximity and then randomly assigned to either the intervention or control group. No other details reported |
| Allocation concealment (selection bias) | High risk | Allocation method not reported |
| Blinding of participants and personnel (performance bias) | High risk | Not reported |
| Blinding of outcome assessment (detection bias) | High risk | Not reported |
| Incomplete outcome data (attrition bias) | Low risk | Loss to follow-up < 20% |
| Selective reporting (reporting bias) | Low risk | Protocol published: "Methodological overview of a five-country community-level HIV/sexually transmitted disease prevention trial" |
| Failure to control for confounders | Low risk | Model adjusted for socio-demographics and non-independence of data |

***Eze, 2020***

| **Bias** | **Authors' judgement** | **Support for judgement** |
| --- | --- | --- |
| Random sequence generation (selection bias) | High risk | Not a randomized controlled trial |
| Allocation concealment (selection bias) | High risk | Not a randomized controlled trial |
| Blinding of participants and personnel (performance bias) | High risk | Not a randomized controlled trial |
| Blinding of outcome assessment (detection bias) | Low risk | Blinding of outcome assessment reported |
| Incomplete outcome data (attrition bias) | High risk | Not a randomized controlled trial |
| Selective reporting (reporting bias) | High risk | Protocol not reported |
| Failure to control for confounders | Low risk | Outcomes not adjusted but no difference between groups identified |

***L'Engle, 2014***

| **Bias** | **Authors' judgement** | **Support for judgement** |
| --- | --- | --- |
| Random sequence generation (selection bias) | Low risk | Random permuted blocks method used in SAS |
| Allocation concealment (selection bias) | Low risk | Assignments concealed in opaque envelopes |
| Blinding of participants and personnel (performance bias) | High risk | Participants and personnel not blinded |
| Blinding of outcome assessment (detection bias) | Low risk | Assessors blinded |
| Incomplete outcome data (attrition bias) | Low risk | Loss to follow-up < 20% |
| Selective reporting (reporting bias) | High risk | No protocol available; changed planned outcome from AUDIT to frequency |
| Failure to control for confounders | Low risk | Outcomes adjusted for HIV status and project site |

***Mertens, 2014***

| **Bias** | **Authors' judgement** | **Support for judgement** |
| --- | --- | --- |
| Random sequence generation (selection bias) | High risk | Though randomized, randomization method not reported |
| Allocation concealment (selection bias) | Low risk | Concealed envelope |
| Blinding of participants and personnel (performance bias) | High risk | Single blind, interviewers blinded only |
| Blinding of outcome assessment (detection bias) | High risk | Blinding of outcome assessment not reported |
| Incomplete outcome data (attrition bias) | Low risk | Loss to follow-up < 20% |
| Selective reporting (reporting bias) | High risk | Protocol not reported |
| Failure to control for confounders | Low risk | All analyses were replicated controlling for baseline variables on which the non-respondents differed |

***Papas, 2020***

| **Bias** | **Authors' judgement** | **Support for judgement** |
| --- | --- | --- |
| Random sequence generation (selection bias) | Low risk | Randomized by computer software, unclear in reported conference abstract but confirmed with study author |
| Allocation concealment (selection bias) | Low risk | Concealed envelope, unclear in reported conference abstract but confirmed with study author |
| Blinding of participants and personnel (performance bias) | High risk | Not blinded, unclear in reported conference abstract but confirmed with study author |
| Blinding of outcome assessment (detection bias) | High risk | Not blinded, unclear in reported conference abstract but confirmed with study author |
| Incomplete outcome data (attrition bias) | Low risk | Loss to follow-up < 20% |
| Selective reporting (reporting bias) | Low risk | Cultural adaption of protocol reported |
| Failure to control for confounders | Unclear risk | Not reported; judged as unclear and not high risk because this was extracted data sent by the study authors |

***Peltzer, 2006***

| **Bias** | **Authors' judgement** | **Support for judgement** |
| --- | --- | --- |
| Random sequence generation (selection bias) | High risk | Not a randomized controlled trial |
| Allocation concealment (selection bias) | High risk | Not a randomized controlled trial |
| Blinding of participants and personnel (performance bias) | High risk | Not a randomized controlled trial |
| Blinding of outcome assessment (detection bias) | High risk | Blinding of outcome assessment not reported |
| Incomplete outcome data (attrition bias) | High risk | Not a randomized controlled trial, pre-test/post-test of BAC taken but not with the same sample of participants |
| Selective reporting (reporting bias) | High risk | No protocol reported |
| Failure to control for confounders | High risk | Outcomes not adjusted |

***Peltzer, 2013***

| **Bias** | **Authors' judgement** | **Support for judgement** |
| --- | --- | --- |
| Random sequence generation (selection bias) | Low risk | Stratified random sampling of clinics conducted using secure remote service |
| Allocation concealment (selection bias) | Low risk | Third party remotely randomized |
| Blinding of participants and personnel (performance bias) | High risk | Participants and clinic staff were not blinded |
| Blinding of outcome assessment (detection bias) | Low risk | Assessors blinded |
| Incomplete outcome data (attrition bias) | High risk | Loss to follow-up > 20% |
| Selective reporting (reporting bias) | Low risk | Protocol available |
| Failure to control for confounders | Low risk | Outcomes adjusted for cluster and for age, sex, and baseline alcohol use disorders identification test score |

***Pengpid, 2013a***

| **Bias** | **Authors' judgement** | **Support for judgement** |
| --- | --- | --- |
| Random sequence generation (selection bias) | Low risk | Computer generated random sequence |
| Allocation concealment (selection bias) | Low risk | Sequentially numbered opaque sealed envelopes |
| Blinding of participants and personnel (performance bias) | High risk | Participants and staff not blinded |
| Blinding of outcome assessment (detection bias) | Low risk | Outcome assessors blinded |
| Incomplete outcome data (attrition bias) | High risk | Loss to follow-up > 20% |
| Selective reporting (reporting bias) | Low risk | Protocol available |
| Failure to control for confounders | Low risk | Analyses controlling for baseline scores, participant gender, age, education, and marital status |

***Pengpid, 2013b***

| **Bias** | **Authors' judgement** | **Support for judgement** |
| --- | --- | --- |
| Random sequence generation (selection bias) | High risk | Randomization method not described |
| Allocation concealment (selection bias) | High risk | Allocation method not described |
| Blinding of participants and personnel (performance bias) | High risk | Participants and staff not blinded |
| Blinding of outcome assessment (detection bias) | Low risk | Outcome assessors blinded |
| Incomplete outcome data (attrition bias) | Low risk | Loss to follow-up < 20% |
| Selective reporting (reporting bias) | Low risk | Protocol available |
| Failure to control for confounders | Low risk | Adjusted for baseline gender and alcohol use |

***Rendall-Mkosi, 2013***

| **Bias** | **Authors' judgement** | **Support for judgement** |
| --- | --- | --- |
| Random sequence generation (selection bias) | Low | Computer random number generator |
| Allocation concealment (selection bias) | High | Sealed envelopes used |
| Blinding of participants and personnel (performance bias) | High | Fieldworkers not blinded |
| Blinding of outcome assessment (detection bias) | High | Not described |
| Incomplete outcome data (attrition bias) | High | Loss to follow-up > 20% |
| Selective reporting (reporting bias) | High | Protocol not available |
| Failure to control for confounders | High | Not adjusted |

***Rotheram-Borus, 2019***

| **Bias** | **Authors' judgement** | **Support for judgement** |
| --- | --- | --- |
| Random sequence generation (selection bias) | High | "Neighborhoods were randomly assigned by UCLA, organized in six blocked sets of four neighborhoods apiece, into 12 intervention neighborhoods and 12 control neighborhoods." How random selection occurred is not reported |
| Allocation concealment (selection bias) | High | Not reported and cluster controlled randomized trial |
| Blinding of participants and personnel (performance bias) | High | Blinding not reported |
| Blinding of outcome assessment (detection bias) | High | Blinding of assessor not described |
| Incomplete outcome data (attrition bias) | Low | Loss to follow-up < 20% at each follow-up |
| Selective reporting (reporting bias) | Low | Trial (NCT00996528) was registered October 15, 2009 at ClinicalTrials.gov |
| Failure to control for confounders | High | Outcomes not adjusted |

***Wechsberg, 2019***

| **Bias** | **Authors' judgement** | **Support for judgement** |
| --- | --- | --- |
| Random sequence generation (selection bias) | Low risk | Computer-generated sequence used to randomize at the cluster level |
| Allocation concealment (selection bias) | High risk | Not reported; cluster randomized |
| Blinding of participants and personnel (performance bias) | High risk | Not blinded |
| Blinding of outcome assessment (detection bias) | High risk | Assessor not blinded |
| Incomplete outcome data (attrition bias) | Low risk | Loss to follow-up < 20% |
| Selective reporting (reporting bias) | High risk | No protocol reported |
| Failure to control for confounders | Low risk | Adjusted for baseline HIV status, sex worker status, and the baseline level of each outcome |
